# Development and validation of the Post-COVID Symptom Scale for Children/Youth (PCSS-C/Y)

**DOI:** 10.1007/s00431-024-05913-9

**Published:** 2024-12-13

**Authors:** Winnie Wan Yee Tso, Yuliang Wang, Daniel Yee Tak Fong, Mike Yat Wah Kwan, Patrick Ip, Jasper Fuk Woo Chan, Lok Kan Leung, Jason Ying Kuen Chan, Sabrina Siu Ling Tsao, Christy Shuk Kuen Chau, Ka Man Yip, Ka Yi Hui, Jaime Sou Rosa Duque, Yu Lung Lau, Tatia Mei Chun Lee

**Affiliations:** 1 Department of Paediatrics and Adolescent Medicine, The University of Hong Kong, Pokfulam, Hong Kong, China; 2 State Key Laboratory of Brain and Cognitive Sciences, The University of Hong Kong, Pokfulam, Hong Kong, China; 3 Department of Paediatrics and Adolescent Medicine, Duchess of Kent Children’s Hospital, Sandy Bay, Hong Kong, China; 4 Department of Paediatrics and Adolescent Medicine, Hong Kong Children’s Hospital, Kowloon Bay, Hong Kong, China; 5 Department of Psychology, The University of Hong Kong, Pokfulam, Hong Kong, China; 6 School of Nursing, The University of Hong Kong, Pokfulam, Hong Kong, China; 7 Department of Paediatrics and Adolescent Medicine, Princess Margaret Hospital, Hong Kong, China; 8 State Key Laboratory of Emerging Infectious DiseasesDepartment of Microbiology, School of Clinical MedicineFaculty of Medicine, Carol Yu Centre for InfectionLi Ka ShingThe University of Hong Kong, Pokfulam, Hong Kong, China; 9 Centre for Virology, Vaccinology and Therapeutics, Hong Kong Science and Technology Park, Sha Tin, Hong Kong, China; 10 Department of Infectious Diseases and Microbiology, The University of Hong Kong‐Shenzhen Hospital, Shenzhen, Guangdong China; 11 Department of Otorhinolaryngology, Head and Neck Surgery, The Chinese University of Hong Kong, Sha Tin, Hong Kong, China

**Keywords:** Post-COVID-19 condition, Symptom burden, Children, Adolescents, Quality of life

## Abstract

This study aims to develop and validate the Post-COVID Symptom Scale for Children/Youth (PCSS-C/Y), which is a comprehensive tool for measuring the symptom burden of post-COVID-19 conditions—persistent symptoms after SARS-CoV-2 infection, commonly known as Long COVID—and its impact on health-related quality of life among children and adolescents. Parents of children and adolescents, adolescents, and young adults with and without a history of COVID-19 were invited to fill in a questionnaire from October 2022 to June 2023. There were 386 valid parent proxy-reported responses, 433 valid adolescent self-reported responses, and 324 valid young adult self-reported responses included in the final analysis. The PCSS-C/Y demonstrated stable factor structure and good internal consistency in different sampling groups. The scale score was negatively associated with Paediatric Quality of Life Inventory (PedsQL) scores (young adult self-report, adjusted *R*^2^ = 0.394; adolescent self-report, adjusted *R*^2^ = 0.219; parent-report, adjusted *R*^2^ = 0.292), while it was positively associated with Strengths and Difficulties Questionnaire (SDQ) scores (young adult self-report, adjusted *R*^2^ = 0.195; adolescent self-report, adjusted *R*^2^ = 0.154; parent-report, adjusted *R*^2^ = 0.239). The scale can also discriminate the post-infected cases and control cases, Cohen’s *d* = 0.41, 0.50, and 0.38 for adult self-report, adolescent self-report, and parent-report, respectively.

*Conclusions*: The PCSS-C/Y is a valid and reliable tool for quantifying the diverse symptomatology of post-COVID-19 conditions in children and adolescents. It provides quantifiable measurements that enable clinicians to monitor post-COVID-19 symptoms in children and young people and facilitates the development of interventions for post-COVID-19 conditions.

**Supplementary Information:**

The online version contains supplementary material available at 10.1007/s00431-024-05913-9.

## Introduction

The coronavirus disease 2019 (COVID-19) pandemic caused by severe acute respiratory syndrome coronavirus 2 (SARS-CoV-2) has affected the health and well-being of individuals of all ages and has so far caused over 6 million deaths worldwide [1]. Although successful vaccination programs have decreased mortality due to COVID-19, many patients who have recovered from acute COVID-19 still suffer from post-COVID-19 conditions. Persistent post-COVID-19 symptoms include fatigue, shortness of breath, and cognitive dysfunction that impact everyday functioning—commonly known as ‘Long COVID’.

Although the majority of children and young people only have mild symptoms after SARS-CoV-2 infection, a recent review estimated that 10 to 20% of children and adolescents had paediatric post-acute sequelae of SARS-CoV-2 (PASC) [2]. Given that over 75% of children and adolescents have been infected with the SARS-CoV-2 virus [3], millions of children worldwide might still be suffering from post-COVID-19 conditions, which can lead to significant burdens on health and society.

There is currently no consensus on the definition of post-COVID-19 symptoms in children and adolescents. The Centre for Disease Control and Prevention (CDC) in the USA defines post-COVID-19 symptoms as signs, symptoms, and conditions that continue or develop after SARS-CoV-2 infection, which are present 4 weeks or more after the initial phase of the infection [4]. The National Institute for Health and Care Excellence (NICE) in the UK defines post-COVID-19 symptoms as signs and symptoms that continue for more than 12 weeks and are not explained by an alternative diagnosis. However, it states that post-COVID-19 syndrome may be considered before 12 weeks while being assessed for alternative underlying disease. NICE has published a set of guidelines for the management of adults, children, and young people who have new or ongoing symptoms 4 weeks or more after the start of acute COVID-19 [5]. The World Health Organization (WHO) has provided a clinical definition of post-COVID-19 conditions in children and adolescents with a history of confirmed or probable SARS-CoV-2 infection based on expert consensus. It defines ‘post-COVID-19 condition’ as symptoms that last at least 2 months, initially occurring within 3 months of acute COVID-19 and impacting everyday functioning [6]. This consensus statement also includes 21 non-specific symptoms that can potentially manifest in children with post-COVID-19 conditions. Moreover, there are variations in the reporting of post-COVID-19 symptoms in children. Existing studies have reported a wide range of estimates on the prevalence of post-COVID-19 symptoms in children ranging from 4 to 46%. Recent studies have also reported that the symptoms appeared to be highly prevalent among non-infected control subjects [7, 8]. One study found a tendency toward better quality of life scores related to emotional and social functioning in cases compared to controls in older children [8]. Existing studies also reported a wide range of symptoms in children and adolescents with post-COVID-19 conditions [7–9]. Hence, it remains uncertain how the persistent but non-specific post-COVID-19 symptoms might affect children and adolescents after SARS-CoV-2 infection.

Due to the lack of empirical evidence in children and adolescents, WHO recommends that more data is needed to better understand post-COVID-19 symptoms in the paediatric population. However, few scales have been developed and validated for assessing the symptom burden of post-COVID-19 conditions in the paediatric population. The Symptom Burden Questionnaire for Long COVID (SBQ-LC) developed by Hughes et al. (2022) is a symptom burden scale for post-COVID-19 conditions in adults [10]. However, SBQ-LC might not be applicable to the paediatric population, because children and young people might experience different post-COVID-19 symptoms to that of the adult population [11]. To address the need for a quantitative measurement of symptom burden in children and adolescents with post-COVID-19 symptoms, we developed and validated the Post-COVID Symptoms Scale (PCSS-C/Y) in a cohort of children/adolescents with or without a history of SARS-CoV-2 infection. Using this scale, we aimed to quantify the impact of the Long COVID symptom burden on children’s overall health-related quality of life. Given that the SARS-CoV-2 virus might eventually become a seasonal coronavirus [12], the creation of this screening tool could assist parents and healthcare professionals in identifying the risk of long-term symptoms after SARS-CoV-2 infection. To this end, we used PCSS-C/Y to assess post-COVID-19 symptomatology in a cohort of children, adolescents, and young adults. We also examined the factor structure, internal consistency, discriminate validity, and clinical validity of PCSS-C/Y.

## Method

### Study population

This was a population-based study using an online questionnaire to assess the symptom burden of post-COVID-19 conditions in children with a history of mild COVID-19 compared to those without a history of COVID-19. The questionnaire was distributed using Qualtrics, which is an online survey software program. Parents of children aged 4–18 years were invited by their schools to join this study and to fill in the questionnaire reporting their children’s post-COVID-19 symptoms and well-being. Meanwhile, adolescents and young adults between 12 and 24 years were also invited to fill in a self-report questionnaire reporting their post-COVID-19 symptoms and well-being. This study was approved by the ethics committee of the Institutional Review Board of the Hong Kong University/Hospital Authority Hong Kong West Cluster and Hospital Authority Central Institutional Review Board (Central IRB) (UW 20–292, UW 20–177, HKCH-REC-2022–009).

Between October 2022 and June 2023, the parent-report questionnaire was distributed to principals and parent groups at kindergartens, primary schools, and secondary schools in the five main districts in Hong Kong (Hong Kong Island, Kowloon East, Kowloon West, New Territories East, and New Territories West), while the self-report questionnaire was distributed to principals and parent groups at secondary schools and to the community. In Hong Kong, preschool children aged 3–6 years attend kindergarten. School-aged children aged 6 to 12 years attend primary school; adolescents aged 13 to 18 years attend secondary school. All parents and self-report participants were asked to give their informed consent and provide their demographic information. Potential duplicate entries or incomplete responders were removed. Children/adolescents outside the target age range were excluded from the analysis. Children with chronic illnesses or genetic disorders and children and adolescents with special educational needs (SEN) including physical disabilities, intellectual disabilities, visual impairment, hearing impairment, and psychiatric disorders were also excluded.

### Development of the PCSS-C/Y

Based on the most prevalent symptoms highlighted in existing studies [6, 8, 13] and consensus from international bodies [4–6] and from a local expert panel (comprising of paediatric infectious disease expert, immunologist, neurologist, respirologist, cardiologist, developmental-behavioural paediatrician/rehabilitation specialist, general Post-COVID Symptom Scale for Children/Youth peadiatrician, neuropsychologist, child psychiatrist, and an ENT surgeon), we selected 17 post-COVID-19 symptoms most likely to cause impaired daily functioning in children and/or adolescents. These 17 symptoms were divided into four sub-domains and used as the basis for the development of PCSS-C/Y. The proposed PCSS-C/Y consisted of 17 items across four domains: (1) neurocognitive/brain fog symptoms (4 items), (2) cardiorespiratory symptoms (4 items), (3) olfactory symptoms (2 items), and (4) non-specific somatic symptoms (7 items). These items were scored on a 5-point Likert scale (0 = never to 4 = always). Participants with a history of mild COVID-19 (either self-reported or parent-reported) were asked to rate the frequency and severity of the symptoms lasting for at least 4 weeks since the initial phase of COVID-19 infection (7 days). Post-COVID Symptom Scale for Children/Youth (PCSS-C/Y) includes two forms: (1) a parent-report form for children aged 4 to 18 years, i.e. “Post-COVID Symptom Scale–Children (PCSS-C)” and (2) a self-report form for youth aged 12 to 24 years, i.e. “Post-COVID Symptom Scale–Youth (PCSS-Y)”. A sample question for PCSS-C was that “After your child has recovered from COVID-19 (starting seven days from the date of infection), please rate the extent to which the following persistent symptoms have affected your child. (Persistent symptoms are defined as those lasting for at least four weeks or more”. The non-infected control participants were asked to report their symptom severity in the last month. The items of PCSS-C/Y are listed in Table S1. The development process of the PCSS-C/Y is shown in supplementary file 2.

The originally developed PCSS-C/Y-20 had 20 items, but there were no significant differences between the post-COVID-19 participants and control participants in neuropsychiatric measures across all three sub-cohorts. As neuropsychiatric symptoms were not more frequently reported in post-COVID-19 cases compared to control subjects, the three items for neuropsychiatric symptoms were excluded from the current PCSS-C/Y scale. For the psychometric parameters of the original PCSS-C/Y-20, see supplementary file 3.

### Other measurements

Health-related quality of life was assessed using the Paediatric Quality of Life Inventory (PedsQL) [14]. Children’s behavioural and emotional difficulties were assessed using the Strengths and Difficulties Questionnaire (SDQ) [15]. Demographic information, including family household income, where they lived, parents’ education level, and whether they were in receipt of social benefits, was obtained from the participants.

### Scale construction and evaluation procedures

An exploratory factor analysis (EFA) was conducted to explore the factor structure of PCSS-C/Y and whether it aligns with the hypothesized symptom domains. As the scale was designed based on the four domains of symptoms, confirmatory factor analysis was performed to examine the factor loading structure of the proposed PCSS-C/Y. In this study, we used root-mean-square error of approximation (RMSEA), Tucker-Lewis Index (TLI), and Comparative Fit Index (CFI) to evaluate the factor structure, according to the guidelines by Hu and Bentler (1998) [16]. We considered an excellent fit as indicated by CFI ≥ 0.95, TLI ≥ 0.93, and RMSEA ≤ 0.06 and a good fit as indicated by CFI ≥ 0.90, TLI ≥ 0.88, and RMSEA ≤ 0.1. Reliability as a measure of internal consistency was assessed by Cronbach’s coefficient alpha. Correlational analyses were conducted to assess various indices of validity. The alpha level for statistical significance was set at 0.05. To interpret the significance of the correlations, we used Cohen’s (1988) criteria:* r* = 0.10 ~ 0.4 (small), 0.3 ~ 0.5 (moderate), > 0.5 (large).

### Validity measures

#### Known-group validity

Given that the prevalence of post-COVID-19 symptoms is small and that the severity of post-COVID-19 symptoms is reported to be mild, we envisage that the current scale can discriminate between the infected sample and control sample in terms of sub-domains and total post-COVID-19 symptom score with a Cohen’s *d* > 0.3.

#### Clinical validity

The clinical validity of PCSS-C/Y was determined by its correlation with PedsQL and SDQ. We recruited a sub-sample of participants to conduct a series of neurocognitive assessments including digital span task [17] and Conners’ continuous performance task [18]. We also administered an additional Sino-Nasal Outcome Test (SNOT-22) Questionnaire [19] to validate the olfactory sub-component of PCSS-C/Y.

### Statistical method

Statistical analyses were performed using SPSS 28.0, SPSS AMOS, and R 4.1.0. Independent sample *t*-test was performed to assess discriminant validity, and a generalized linear model was used to evaluate the validity of PCSS-C/Y, with PCSS-C/Y or its sub-component inputted as the predictor, validation scales inputted as a criterion, and age and gender inputted as covariates. Partial *R*-square was used to evaluate the effect size of the correlation between PCSS-C/Y and the validation measurements.

## Results

### Sample characteristics

A total of 490 parents and 796 adolescents/young adults filled in the questionnaire from October 2022 to June 2023. After examining the data integrity, 386 valid parent-reported responses, 433 valid adolescent self-reported responses, and 324 young adult self-reported responses were included in the final analysis. Of the excluded responses, 53 had SEN or chronic illness, 78 had substantial missing values, 23 were duplicates, and 53 had invalid responses that were deemed as careless/insufficient effort (C/IE) responders (e.g. abnormal speed of response, invariability as indicated in the long string analysis [20]) (Fig. S1a). In the included dataset, 278 young adults (85.8%) and 274 adolescents (63.3%) self-reported that they had had a SARS-CoV-2 infection, while 245 parents (72.9%) reported that their children had had a SARS-CoV-2 infection. For participants with previous COVID-19 infections, the time since infection ranged from 36 to 161 days, with a median period of 54 (IQR = 31) days post-infection (see Fig. S1b). There were no significant differences between the demographic details of the participants (Table 1). The following results are based on the responses from infected cases, except for the discriminant validity analysis that compared the responses between infected cases and control subjects.

**Table 1:** Demographic details of the included subjects

	Young adult self-report (N = 324)			Adolescent self-report (*N* = 433)			Parent-report (*N* = 386)		
Age ranges (years)	19–24			12–18			4–17		
	Infected cases (*N* = 278)	Non-infected controls (*N* = 46)	*p*	Infected cases (*N* = 274)	Non-infected controls (*N* = 159)	*p*	Infected cases (*N* = 245)	Non-infected controls (*N* = 141)	*p*
	Mean/*N* (SD/%)	Mean/*N* (SD/%)		Mean/*N* (SD/%)	Mean/*N* (SD/%)		Mean/*N* (SD/%)	Mean/*N* (SD/%)	
Age (years)	21.78 (2.51)	21.36 (1.65)	0.280	15.17 (2.00)	14.91 (1.93)	0.862	10.50 (3.87)	10.98 (3.48)	0.210
Females	189 (68)	33 (72)	0.613	151 (55.1)	89 (55.6)	0.190	130 (53.1)	70 (49.6)	0.471
Non-Chinese	0 (0.0)	0 (0.0)	1.000	2 (0.7)	1 (0.6)	0.874	0 (0.0)	1 (0.7)	0.319
Vaccination status^a^									
Never	44 (15.8)	0 (0.0)		37 (13.5)	5 (3.1)		22 (8.9)	7 (5.0)	
1 dose	4 (1.4)	0 (0.0)		19 (6.9)	2 (1.3)		33 (13.5)	4 (2.8)	
2 doses	84 (30.2)	2 (4.3)		107 (39.1)	47 (29.6)		104 (42.4)	27 (19.1)	
3 doses or above	146 (52.5)	44 (95.7)		111 (40.5)	105 (66.0)		86 (35.1)	103 (73.0)	
BMI	20.67 (2.84)	20.01 (2.05)	0.142	20.77 (5.68)	20.20 (3.94)	0.279	17.08 (5.46)	17.89 (7.76)	0.118
Family SES^b^	−0.07 (0.96)	0.15 (1.00)	0.190	−0.03 (0.87)^c^	0.07 (1.26)	0.420	−0.02 (0.99)	0.03 (1.02)	0.812

^a^Infected subjects’ vaccination status is determined by the number of doses received 14 days prior to their SARS-CoV-2 infection. Control subjects’ vaccination status is determined by the number of doses received 14 days prior to their study participation

^b^Family socioeconomic status (SES) was determined using a composite *Z*-score calculated from a principal component analysis (PCA) of multiple factors, including family monthly income, per capita household living area, parents’ education levels, and the recipient status of social benefits

^c^Adolescent report family SES (*Z*-score) was estimated based on per capita household living area

### Exploratory factor analysis

An exploratory factor analysis (EFA) was conducted using the maximum likelihood extraction method, retaining factors with eigenvalues greater than 1. Varimax rotation with Kaiser normalization was applied. The EFA results indicated a four-factor solution across all three age groups (see Table S2a-c). This factor structure supports our hypothesized symptom domains.

### Confirmatory factor analysis

**Fig. 1:**
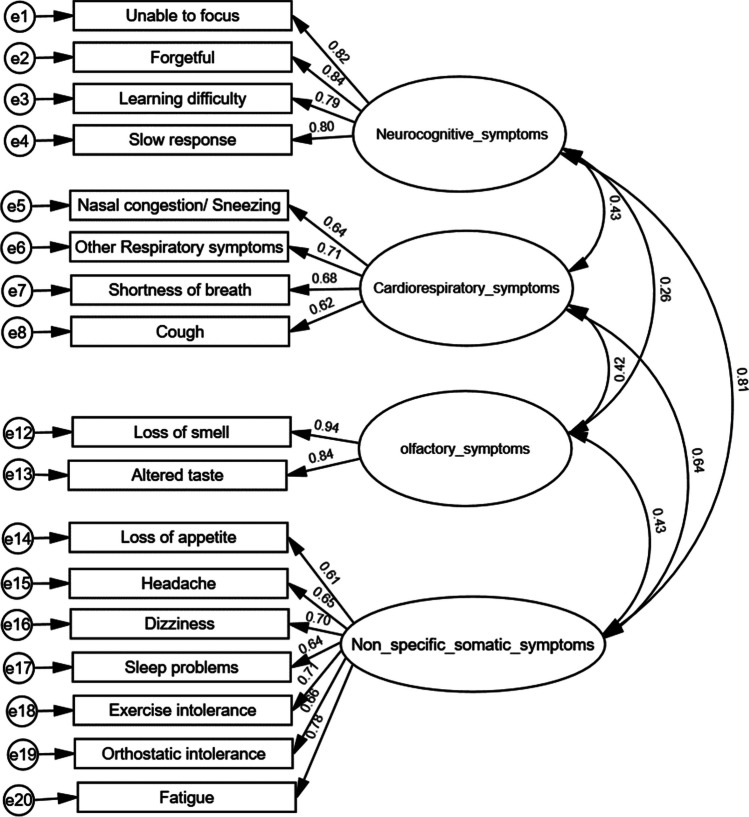

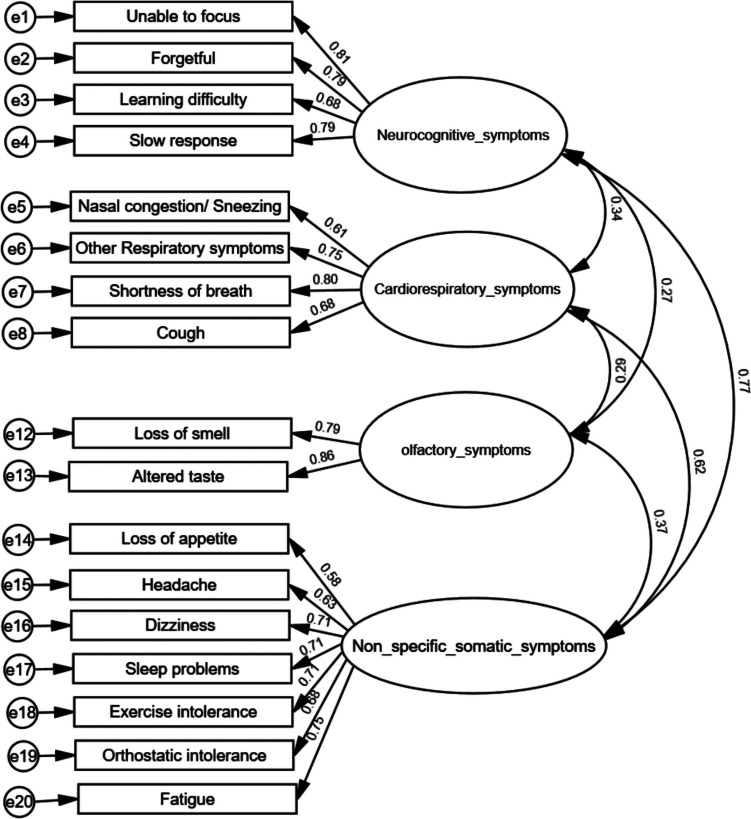

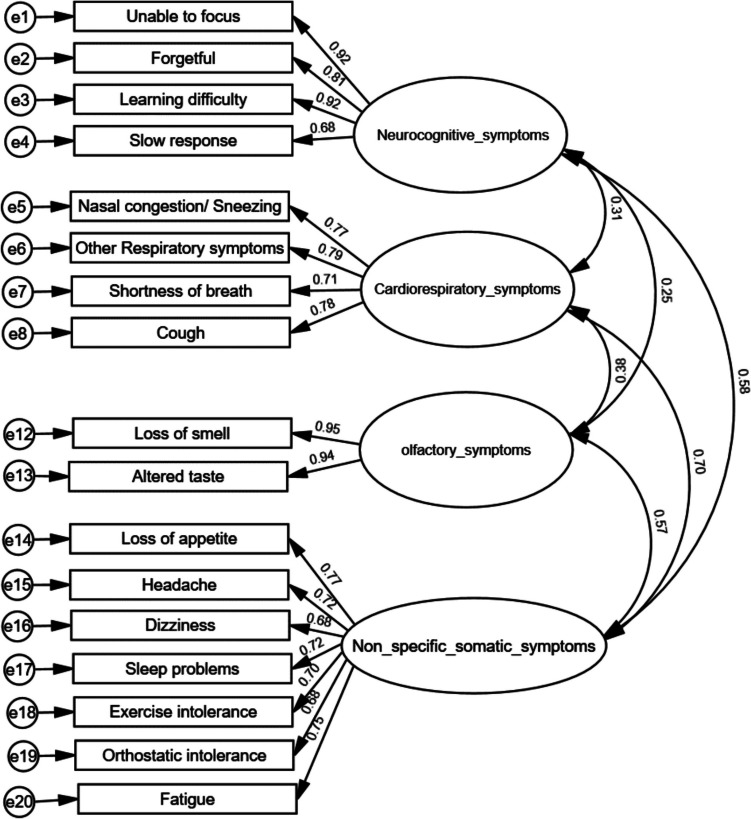
**Aa** Factor loading properties of self-report PCSS-Y by young adults (age between 19 and 24), CFI = 0.93, TLI = 0.91, RMSEA = 0.072, *N* = 278. **b** Factor loading properties of self-report PCSS-Y by adolescents (age between 12 and 18), CFI = 0.91, TLI = 0.89, RMSEA = 0.079, *N* = 274. **c** Factor loading properties of parent-report PCSS-C, CFI = 0.90, TLI = 0.88 and RMSEA = 0.100, *N* = 245

The hypothesis-based four-factor model was examined by cases at different age ranges, respectively (Fig. 1a–c). The young adult self-report PCSS-Y model demonstrated a good fit (CFI = 0.93, TLI = 0.91, and RMSEA = 0.072) (Fig. 1a). The adolescent self-report PCSS-Y model also demonstrated a good fit (CFI = 0.91, TLI = 0.89, and RMSEA = 0.079) (Fig. 1b). The parent-report PCSS-C model demonstrated a fair fit (CFI = 0.90, TLI = 0.88, and RMSEA = 0.100) (Fig. 1c).

### Internal consistency

Cronbach’s alpha coefficient was 0.91 for parent-report PCSS-C, 0.89 for adolescent self-report PCSS-Y, and 0.91 for young adult self-report PCSS-Y, indicating good internal consistency. For the sub-domains, the internal consistency ranged between 0.76 and 0.89 for the self-report PCSS-C/Y and 0.81 and 0.94 for the parent-report PCSS-C. For item-based internal consistency coefficients, see Table 2.

**Table 2:** Internal consistency (Cronbach’s alpha) of the paediatric Post-COVID Symptom Scale (PCSS)

	Self-report PCSS-Y		Parent-report PCSS-C
	Young adults (*N* = 278)	Adolescents (*N* = 274)	Children and adolescents (*N* = 245)
Neurocognitive	0.89	0.85	0.85
Cardiorespiratory	0.76	0.80	0.81
Olfactory	0.88	0.81	0.94
Non-specific Somatic symptoms	0.86	0.86	0.88
Whole scale	0.90	0.89	0.91

### Known-group validity

Infected cases scored significantly higher on the PCSS-C/Y when compared to the control cases (Table 3), with Cohen’s *d* of 0.41, 0.50, and 0.38 for adult self-report, adolescent self-report, and parent-report PCSS-C/Y, respectively. Significant differences were observed for all domains in PCSS-C/Y across the three sub-cohorts, with the standardized difference (Cohen’s *d*) among the different subgroups ranging from 0.19–0.27 in the neurocognitive sub-domain, 0.34–0.80 in the cardiorespiratory sub-domain, 0.35–0.50 in the olfactory sub-domain, and 0.33–0.39 in the non-specific somatic symptom sub-domain. Long COVID symptoms between infected and non-infected subjects in parent-report PCSS-C for children below 12 years are demonstrated in Table S3. The item-based discriminative ability for comparing those infected to those never infected is illustrated in Fig. S2(a-c).

**Table 3:** Comparison of PCSS-C/Y scores between infected cases and non-infected controls

	Young adult self-report (278 vs. 46)				Adolescent self-report (274 vs. 159)				Parent-report (245 vs. 141)			
	*t*	Two-sided *p*-value	FDR-adjusted *p*-value	Cohen’s *d*	*t*	Two-sided *p*-value	FDR-adjusted *p*-value	Cohen’s *d*	*t*	Two-sided *p*-value	FDR-adjusted *p*-value	Cohen’s *d*
Neurocognitive	2.067	0.042	0.042	0.27	2.009	0.045	0.045	0.19	1.998	0.037	0.037	0.20
Cardiorespiratory	6.278	< 0.001	< 0.001	0.80	7.219	< 0.001	< 0.001	0.65	3.427	< 0.001	0.002	0.34
Olfactory	5.820	< 0.001	< 0.001	0.50	5.326	< 0.001	< 0.001	0.44	3.631	< 0.001	0.002	0.35
Non-specific somatic symptoms	2.472	0.002	0.003	0.39	3.631	< 0.001	< 0.001	0.35	3.143	0.002	0.003	0.33
Whole scale	4.542	< 0.001	0.001	0.60	5.454	< 0.001	< 0.001	0.50	3.820	< 0.001	0.001	0.38

*FDR* false discovery rate

### Clinical validity

The construct validity of the PCSS-C/Y was supported by multiple analyses across the subscales and various demographics. The total scores from PCSS-C/Y demonstrated significant negative correlations with PedsQL total scores (young adult self-report, *R*^2^ = 0.394; adolescent self-report, *R*^2^ = 0.219; parent-report, *R*^2^ = 0.292) and positive correlations with SDQ total scores (young adult self-report, *R*^2^ = 0.195; adolescent self-report, *R*^2^ = 0.154; parent-report, *R*^2^ = 0.239), all with FDR-adjusted *p*-values of < 0.001, indicating that the higher PCSS-C/Y scores were associated with poorer quality of life and greater difficulties (Table 4). The neurocognitive subscale showed significant negative correlations with neurocognitive tests including Digital Span in the parent-report (*R*^2^ = 0.174, FDR-adjusted *p*-value = 0.028) and CPT-3 D-prime in young adult self-report (*R*^2^ = 0.166, FDR-adjusted *p*-value = 0.018) (Table S4a). The cardiorespiratory subscale showed strong negative correlations with the PedsQL physical health subscale (young adult self-report, *R*^2^ = 0.058; adolescent self-report, *R*^2^ = 0.050; parent-report, *R*^2^ = 0.050), all with *p* < 0.001, which underscores its impact on physical health (Table S4b). The olfactory subscale was correlated with the Sino-Nasal Outcome Test (SNOT) scores (self-report, *R*^2^ = 0.142, *p* = 0.025; parent-report, *R*^2^ = 0.336, *p* = 0.021), confirming its utility in detecting olfactory disturbances (Table S4c). Finally, the other somatic symptom subscale showed significant negative correlations with PedsQL (young adult self-report, *R*^2^ = 0.331; adolescent self-report, *R*^2^ = 0.194; parent-report, *R*^2^ = 0.397) and self-perceived health status, all with FDR-adjusted *p*-value < 0.001, emphasizing the broad impact of somatic symptoms (Table S4d). The correlation between PCSS-C/Y and PedsQL and SDQ subscales across the three subgroups is shown in Table S5(a-c). To confirm the current findings, a subgroup analysis was conducted on a total of 153 case and control subjects with confirmatory testing (NP/ORF-8 antibody results), which showed similar findings to the main cohort in terms of the correlation between PCSS-C/Y and PedsQL/SDQ (Table S6).

**Table 4:** Construct validity of PCSS-C/Y (whole scale)

Variables	Young adult self-report PCSS-Y (*N* = 278)				Adolescent self-report PCSS-Y (*N* = 274)				Parent-report PCSS-C (*N* = 245)			
	Estimate (SE)	Two-sided *p*-value	FDR-adjusted *p*-value	*R*^2^	Estimate (SE)	Two-sided *p*-value	FDR-adjusted *p*-value	*R*^2^	Estimate (SE)	Two-sided *p*-value	FDR-adjusted *p*-value	*R*^2^
PedsQL total score	− 0.864 (0.068)	< 0.001	< 0.001	0.395	− 0.795 (0.084)	< 0.001	< 0.001	0.219	− 0.590 (0.059)	< 0.001	< 0.001	0.292
SDQ total score	0.192 (0.025)	< 0.001	< 0.001	0.195	0.197 (0.030)	< 0.001	< 0.001	0.154	0.292 (0.033)	< 0.001	< 0.001	0.239
Self-perceived health status	− 0.548 (0.073)	< 0.001	< 0.001	0.179	− 0.678 (0.082)	< 0.001	< 0.001	0.192	− 0.763 (0.085)	< 0.001	< 0.001	0.248

*PedsQL* Paediatric Quality of Life Inventory, *SDQ* Strengths and Difficulties Questionnaire

^*^Linear regression model was applied using PCSS-C/Y total score as the predictor and PedsQL, SDQ, and self-perceived health status as criterion, respectively, adjusted by age, gender, and SES. Partial *R*-square (*R*^2^) was reported

### Face validity

Participants who had COVID-19 were asked to rate their symptoms compared to pre-COVID-19 (see Fig. S3a-c). For young adult self-report, between 11.8 and 50% of participants rated they had worse symptoms across the 17 items. For adolescent self-report, between 5.5 and 33.6% of participants rated worse symptoms across the 17 items. For parent-report, between 4.3 and 21.3% of parents rated their children had worse symptoms across the 17 items.

### Scoring and cut-offs for PCSS-C/Y

Each PCSS-C/Y item was rated on a 5-point Likert scale (0 = never to 4 = always), and the total score was calculated by summing all the item scores. Individual scores above the 90% and 95% percentile were considered as the cut-off scores indicating moderate risk and high risk for poorer well-being due to post-COVID-19 symptoms (Table S7). The symptom-based characteristics of cases who scored above the 90% and 95% percentile were demonstrated in Fig. S4(a-c).

## Discussion

This study developed and tested the validity of the 17-item PCSS-C/Y for assessing post-COVID-19 symptoms in the paediatric population. We confirmed our hypothesized factor structure of PCSS-C/Y, with CFI greater than 0.9 in all three groups of participants, and such factor structure was identical across the different age groups. We also demonstrated good internal consistency with Cronbach’s alpha higher than 0.89 for each age group and acceptable Cronbach’s alpha > 0.70 for each subscale. The PCSS-C/Y scores (and subscales scores) were highly correlated with gold-standard quality of life measurements and emotional/behavioural problems (PedsQL and SDQ), highlighting its practical value in reflecting functional disturbances and emotional and behavioural issues associated with post-COVID-19 conditions in the paediatric population.

The newly developed PCSS-C/Y enables clinicians or healthcare professionals to quantify and monitor the symptom burden of post-COVID-19 conditions in children and adolescents. Children, adolescents, and college students (young adults) with persistent COVID-19 symptoms for at least 4 weeks with PCSS-C/Y scores above the cut-off are at risk of poorer well-being. The cut-off percentile (5% and 10% for moderate and high risk, respectively) was consistent with that of the estimated prevalence for Long COVID symptoms [21]. At the individual level, PCSS-C/Y can reveal the body system that would be most affected as a result of post-COVID-19 symptoms. This can facilitate referral to the appropriate specialists or healthcare professionals for further assessment and/or rehabilitation.

Our study findings also characterized post-COVID-19 symptoms between children, adolescents, and college students (young adults). Interestingly, our findings demonstrated that post-COVID-19 symptoms vary according to the age group of children and young people. Adolescents and young adults with a history of COVID-19 were found to have significantly more neurocognitive symptoms than those without a history of COVID-19. In young children, there were no significant differences in neurocognitive symptoms between infected and uninfected groups. All three groups did not have significant differences in their neuropsychiatric symptoms compared to uninfected control subjects. These findings were consistent with the findings by Taquet et al. [22], where children with SARS-CoV-2 infection did not have an increased risk of mood or anxiety disorders.

Among the three age groups, the adolescent self-report sample exhibited the most optimal factor structure, internal consistency, and clinical validity, followed by the young adult self-report sample and parent-report sample. Clinically, the SARS-CoV-2 virus might affect people differently depending on their age and developmental stage. It is plausible that neurocognitive symptoms such as forgetfulness, slow response, and inability to focus might be less obvious than physical symptoms (cardiorespiratory, olfactory, and non-specific somatic symptoms). Children and young people might not be aware of their neurocognitive problems until they encounter learning difficulties or deterioration in their academic performance. Therefore, middle school/college students who are noted to have persistently deteriorated learning a few months after an episode of COVID-19 may need to be monitored by educators or school social workers and referred for assessment by a psychologist for learning support. The PCSS-C/Y can also be used to monitor symptom progression/improvement, which can guide the duration of the learning support and the need for medical intervention.

Our study suggested that self-report surveys for adolescents or young adults had a more stable factor structure than the parent-report survey. Because of the mild symptomology of post-COVID-19 conditions in the majority of the infected cases, children/adolescents might not be able to adequately describe their symptoms to their parents/ caregivers; hence, mild post-COVID-19 symptoms might be underreported in proxy-reported surveys. Nevertheless, our preliminary validation metrics indicate that the parent-report version is acceptable. Ongoing clinical and epidemiological studies using the parent-report PCSS-C can further improve the validity of the questionnaire. It is important to note that the discrepancies in the psychometric properties among different age groups could be attributed to variations in sample sizes across the three groups.

## Limitations

Our study has some limitations that need to be addressed. First, this was a population-based survey, and we were unable to obtain laboratory confirmation of COVID-19 diagnosis or antibody testing for all cases and control subjects, respectively. Nevertheless, a subgroup of 153 cases and control subjects were invited to have confirmatory testing. Positive PCR results confirmed SARS-CoV-2 infection in case subjects, whereas negative NP/ORF-8 antibody results confirmed no recent COVID-19 infection in control subjects. Subgroup analysis of the 153 subjects with confirmatory testing showed similar findings to that of the main cohort, confirming that case subjects had higher PCSS-C/Y scores and poorer quality of life. Second, the survey-based study might be prone to recall or reporting bias. However, the electronic survey is the most effective approach to collect data from a large number of subjects within a short period of time to enable accurate reporting of post-COVID-19 symptoms. Third, participants were asked to report their subjective symptoms. To quantify these symptoms, the survey asked specifically whether these symptoms were present prior to COVID-19 infection and. if so, whether there was an improvement or worsening of the symptoms. Despite the participants completing the survey at varying intervals ranging from 1 to 5 months post-infection, no association was observed between the duration since infection and the reported symptoms. We also asked uninfected control subjects to report the same set of symptoms at the time of the survey and whether they had experienced similar symptoms 4 weeks prior. Finally, there were other unaccounted factors that could be responsible for the observed differences in post-COVID-19 symptoms between case and control subjects. The mental well-being of children/adolescents might have been affected by social restrictions and school closures during the pandemic. Our survey was conducted from October 2022 to June 2023. It is noteworthy that the social distancing restrictions were lifted by the Hong Kong Government in December 2022. However, we did not observe a significant difference in self-reported or parent-reported post-COVID-19 symptoms among individuals infected before or after the lifting of the social restrictions. Furthermore, although the survey was conducted after schools re-opened, some children/adolescents might require more time to adapt to the return-to-school arrangements [23].

## Conclusions

To our knowledge, this study is the first to develop and confirm the reliability and validity of a self-reported/proxy-reported scale for post-COVID-19 symptoms in children and young people to measure symptom burden after COVID-19. The PCSS-C/Y is simple and easy to complete and has promising psychometric properties, allowing clinicians to screen for post-COVID-19 symptoms in children and young people. Examining the symptom distribution among the different domains within the PCSS-C/Y can facilitate the design of rehabilitation plans accordingly. It can also be an invaluable tool for paediatric post-COVID-19 research studies.

## Supplementary Information

Below is the link to the electronic supplementary material.

Supplementary file1 (DOCX 886 KB)

Supplementary file2 (DOCX 51 KB)

Supplementary file3 (DOCX 28 KB)

## Data Availability

The dataset will not be made available publicly. Request to access the data should be directed to the corresponding author.
