# Supplementary material for: Development and validation of the Post-COVID Symptom Scale for Children/Youth (PCSS-C/Y)
Source: Eur J Pediatr. 2024 Dec 13;184(1):81. doi: 10.1007/s00431-024-05913-9 (PMC11645425; doi:10.1007/s00431-024-05913-9)
Supplement: Supplementary file 1 — Supplementary file1 (DOCX 886 KB) [file 431_2024_5913_MOESM1_ESM.docx]

**Supplementary Tables**

1. **Supplementary Table S1**: The 17-item Post-COVID Symptom Scale - Children/Youth (PCSS-C/Y)
2. **Supplementary Table S2a**: Exploratory Factor Analysis - PCSS-Y Factor Loadings (Young Adult Self-report)
3. **Supplementary Table S2b**: Exploratory Factor Analysis - PCSS-Y Factor Loadings (Adolescent Self-report)
4. **Supplementary Table S2c**: Exploratory Factor Analysis - PCSS-C Factor Loadings (Parent-report)
5. **Supplementary Table S3**: Comparison of Long COVID Symptoms Between Infected and Non-infected Subjects in Parent-report PCSS-C for Children Below 12 Years
6. **Supplementary Table S4**: Construct Validity of Subscales of PCSS-C/Y
7. **Supplementary Table S5**: Correlations Between PCSS-C/Y and PedsQL and SDQ
8. **Supplementary Table S6**: Correlations Between PCSS-C/Y and PedsQL and SDQ Among Infected Cases Confirmed by NP/ORF-8 Antibody Results
9. **Supplementary Table S7**: Cut-off Scores for PCSS-C/Y with Reference to Mean PedsQL Score

**Supplementary Figures**

1. **Figure S1a**: Data Inclusion Flow Chart
2. **Figure S1b**: Timeline of Infection and Survey Participation for Individuals Infected with COVID-19
3. **Figure S2a**: Standardized Mean Differences in Adolescent Self-report PPCS (Error Bars Represent 95% CI)
4. **Figure S2b**: Standardized Mean Differences in Young Adult Self-report PCSS-Y (Error Bars Represent 95% CI)
5. **Figure S2c**: Comparison of PCSS-C Symptoms in Parent-reported Children and Adolescents Infected with SARS-CoV-2 and Control Subjects (Error Bars Represent 95% CI)
6. **Figure S3a**: Young Adult Self-rating Post-COVID Symptoms Compared to Pre-infection
7. **Figure S3b**: Adolescent Self-rating Post-COVID Symptoms Compared to Pre-infection
8. **Figure S3c**: Parent-reported Post-COVID Symptoms Compared to Pre-infection
9. **Figure S4a**: Young Adult Self-rating Post-COVID Symptoms by Percentile Cut-offs
10. **Figure S4b**: Adolescent Self-rating Post-COVID Symptoms by Percentile Cut-offs
11. **Figure S4c**: Parent-report Post-COVID Symptoms by Percentile Cut-offs

Supplementary Table S1. The 17-item Post-COVID Symptom Scale -Children/Youth (PCSS-C/Y)

| PCSS-C/Y subscales | Items in English | Items in Chinese |
| --- | --- | --- |
| Brain fog/ neurocognitive symptoms | Unable to focus, being easily distracted | 專注力弱,容易分心,不能長 |
|  | Memory issue, forgetful | 記憶力問題、善忘 |
|  | Learning difficulty, cannot keep up with homework | 學習問題,功課跟不上 |
|  | Slow response | 反應較慢 |
|  |  |  |
| Cardiorespiratory symptoms | Nasal congestion/ Sneezing | 鼻塞/ 流鼻水 |
|  | Non-specific Respiratory symptoms (e.g., sore throat, sputum) | 其他上呼吸道症狀(例如:喉嚨痛、有痰) |
|  | Shortness of breath | 呼吸困難/氣促 |
|  | Cough | 咳嗽 |
| Olfactory symptoms | Loss of smell | 嗅覺失靈 |
|  | Altered taste | 味覺失靈 |
| Non-specific somatic symptoms | Loss of appetite | 食慾不振 |
|  | Headache | 頭痛 |
|  | Dizziness | 頭暈 |
|  | Sleep problems (e.g., Insomnia, hypersomnia) | 睡眠問題(例如失眠或嗜睡) |
|  | Exercise intolerance | 體能變差/易攰 |
|  | Orthostatic intolerance (dizziness, weakness or nausea when standing up) | 自律神經失調(蹲下久坐起身時頭暈) |
|  | Fatigue | 疲倦 |

| Table S2a. Exploratory factor analysis: PCSS-Y Factor loadings. Young Adult-self-report | | | | | | | |
| --- | --- | --- | --- | --- | --- | --- | --- |
|  |  |  |  | Factor |  |  |  |
|  |  |  |  | I | II | III | IV |
| Neurocognitive symptoms | | | Unable to focus | **0.806** | 0.129 | 0.134 | 0.010 |
|  |  |  | Forgetful | **0.822** | 0.141 | 0.091 | 0.047 |
|  |  |  | Learning difficulty | **0.747** | 0.094 | 0.129 | 0.116 |
|  |  |  | Slow response | **0.745** | 0.132 | 0.153 | 0.130 |
| Cardiorespiratory symptoms | | | Nasal congestion/ Sneezing | 0.180 | **0.544** | 0.190 | 0.147 |
|  |  |  | Other Respiratory symptoms | 0.147 | **0.701** | 0.123 | 0.156 |
|  |  |  | Shortness of breath | 0.284 | **0.554** | 0.214 | 0.166 |
|  |  |  | Cough | 0.052 | **0.726** | 0.091 | 0.059 |
| Olfactory Symptoms | | | Altered taste | 0.081 | 0.204 | 0.141 | **0.763** |
|  |  |  | Loss of smell | 0.117 | 0.179 | 0.135 | **0.949** |
| Non-Specific Somatic symptoms | | | Loss of appetite | 0.323 | 0.217 | **0.380** | 0.275 |
|  |  |  | Headache | 0.283 | 0.205 | **0.697** | 0.124 |
|  |  |  | Dizziness | 0.287 | 0.259 | **0.722** | 0.168 |
|  |  |  | Sleep problems | 0.282 | 0.117 | **0.540** | 0.084 |
|  |  |  | Exercise intolerance | 0.300 | 0.256 | **0.578** | 0.090 |
|  |  |  | Orthostatic intolerance | 0.369 | 0.130 | **0.537** | 0.045 |
|  |  |  | Fatigue | 0.362 | 0.268 | **0.590** | 0.120 |
| Note: Extraction Method: Maximum Likelihood. | | | | | |  |  |
| Rotation Method: Varimax with Kaiser Normalization. | | | | | | |  |
| Rotation converged in 6 iterations.  Bold = salient loading (>=.38) | | | | | |  |  |

| Table S2b. Exploratory factor analysis: PCSS-Y Factor loadings. Adolescents-self-report | | | | | | | |
| --- | --- | --- | --- | --- | --- | --- | --- |
|  |  |  |  | Factor |  |  |  |
|  |  |  |  | I | II | III | IV |
| Neurocognitive symptoms | | | Unable to focus | **0.767** | 0.030 | 0.126 | 0.066 |
|  |  |  | Forgetful | **0.751** | 0.061 | 0.105 | 0.054 |
|  |  |  | Learning difficulty | **0.647** | 0.078 | 0.104 | 0.021 |
|  |  |  | Slow response | **0.691** | 0.196 | 0.225 | 0.097 |
| Cardiorespiratory symptoms | | | Nasal congestion/ Sneezing | 0.178 | **0.569** | 0.143 | 0.012 |
|  |  |  | Other Respiratory symptoms | 0.125 | **0.663** | 0.175 | 0.184 |
|  |  |  | Shortness of breath | 0.142 | **0.786** | 0.197 | 0.040 |
|  |  |  | Cough | 0.019 | **0.699** | 0.121 | 0.137 |
| Olfactory Symptoms | | | Altered taste | 0.075 | 0.139 | 0.101 | **0.923** |
|  |  |  | Loss of smell | 0.148 | 0.128 | 0.077 | **0.721** |
| Non-Specific Somatic symptoms | | | Loss of appetite | 0.320 | 0.286 | **0.387** | 0.140 |
|  |  |  | Headache | 0.189 | 0.229 | **0.713** | 0.004 |
|  |  |  | Dizziness | 0.247 | 0.208 | **0.776** | 0.138 |
|  |  |  | Sleep problems | 0.352 | 0.200 | **0.472** | 0.113 |
|  |  |  | Exercise intolerance | 0.310 | 0.251 | **0.539** | 0.200 |
|  |  |  | Orthostatic intolerance | **0.413** | 0.178 | **0.476** | 0.098 |
|  |  |  | Fatigue | **0.381** | 0.384 | **0.441** | 0.102 |
| Note: Extraction Method: Maximum Likelihood. | | | | | |  |  |
| Rotation Method: Varimax with Kaiser Normalization. | | | | | | |  |
| Rotation converged in 6 iterations.  Bold = salient loading (>=.38) | | | | | |  |  |

| Table S2c..Exploratory factor analysis PCSS-C: Factor loadings. Parent-report | | | | | | | |
| --- | --- | --- | --- | --- | --- | --- | --- |
|  |  |  |  | Factor |  |  |  |
|  |  |  |  | I | II | III | IV |
| Neurocognitive symptoms | | | Unable to focus | **0.853** | 0.097 | 0.230 | 0.068 |
|  |  |  | Forgetful | **0.759** | 0.065 | 0.255 | 0.041 |
|  |  |  | Learning difficulty | **0.899** | 0.095 | 0.164 | 0.061 |
|  |  |  | Slow response | **0.589** | 0.078 | **0.492** | 0.075 |
| Cardiorespiratory symptoms | | | Nasal congestion/ Sneezing | 0.074 | **0.737** | 0.203 | 0.069 |
|  |  |  | Other Respiratory symptoms | 0.010 | **0.703** | 0.346 | 0.055 |
|  |  |  | Shortness of breath | 0.269 | **0.593** | 0.289 | 0.158 |
|  |  |  | Cough | 0.023 | **0.765** | 0.205 | 0.141 |
| Olfactory Symptoms | | | Altered taste | 0.039 | 0.148 | 0.272 | **0.904** |
|  |  |  | Loss of smell | 0.097 | 0.145 | 0.249 | **0.856** |
| Non-Specific Somatic symptoms | | | Loss of appetite | 0.208 | 0.362 | **0.633** | 0.192 |
|  |  |  | Headache | 0.151 | 0.247 | **0.554** | 0.166 |
|  |  |  | Dizziness | 0.143 | 0.228 | **0.564** | 0.120 |
|  |  |  | Sleep problems | 0.198 | 0.216 | **0.689** | 0.101 |
|  |  |  | Exercise intolerance | 0.179 | 0.180 | **0.689** | 0.233 |
|  |  |  | Orthostatic intolerance | 0.146 | 0.190 | **0.581** | 0.258 |
|  |  |  | Fatigue | 0.209 | 0.373 | **0.653** | 0.081 |
| Note: Extraction Method: Maximum Likelihood. | | | | | |  |  |
| Rotation Method: Varimax with Kaiser Normalization. | | | | | | |  |
| Rotation converged in 6 iterations.  Bold = salient loading (>=.38) | | | | | |  |  |

Supplementary Table S3. Comparison of Long COVID symptoms between infected and non-infected subjects in parent-report PCSS-C for children below 12 years (156 vs. 83)

| PCSS-C Subscales | t | Two-sided p-value | FDR-adjusted p-value | Cohen's d |
| --- | --- | --- | --- | --- |
| Neurocognitive | 1.99 | 0.048 | 0.118 | 0.270 |
| Cardiorespiratory | 1.55 | 0.122 | 0.122 | 0.211 |
| Olfactory | 1.65 | 0.094 | 0.118 | 0.224 |
| Non-specific somatic symptoms | 1.77 | 0.078 | 0.118 | 0.241 |
| Whole PCSS-C | 2.22 | 0.014 | 0.070 | 0.302 |

Supplementary Table S4. Construct validity of subscales of PCSS-C/Y

1. Neurocognitive subscales

|  | Young adult self-report (n =49) | | | | Adolescent self-report subscale (n =31) | | | | Parent-report (children below 12) (n =30) | | | | |
| --- | --- | --- | --- | --- | --- | --- | --- | --- | --- | --- | --- | --- | --- |
| Outcome measures* | Estimate  (se) | Two-sided p-value | FDR-adjusted p-value | R^2^ | Estimate  (se) | Two-sided p-value | FDR-adjusted p-value | R^2^ | Estimate  (se) | Two-sided p-value | FDR-adjusted p-value | R^2^ |  |
| Digital Span | -0.09 (0.14) | 0.570 | 0.760 | 0.033 | -0.33 (0.15) | 0.035 | 0.070 | 0.173 | -0.41  (0.22) | 0.007 | 0.028 | 0.174 |  |
| CPT-3 D-prime | -0.26 (0.09) | 0.009 | 0.018 | 0.166 | -0.14  (0.09) | 0.153 | 0.204 | 0.071 | -0.04  (0.11) | 0.681 | 0.770 | 0.045 |  |
| CPT-3 RT variation | 0.37  (0.11) | 0.001 | 0.004 | 0.208 | 0.18  (0.07) | 0.015 | 0.060 | 0.192 | 0.02  (0.07) | 0.770 | 0.770 | 0.047 |  |
| CPT -3 Hit RT | 0.01  (0.01) | 0.995 | 0.995 | 0.026 | 0.01  (0.01) | 0.467 | 0.467 | 0.040 | 0.01  (0.01) | 0.394 | 0.770 | 0.068 |  |

*Linear regression model was applied using PCSS-C/Y neurocognitive subscale as the predictor and Neurocognitive measures (digital span, CPT-3 D-prime, CPT-3 Reaction Time variation, CPT-3 Hit Reaction Time) as criterion, respectively, with age and gender inputted as covariates.

1. Cardiorespiratory subscale

|  | Young adult self-report (n =278) | | | Adolescent self-report (n =274) | | | Parent-report cardiorespiratory subscale (n = 245) | | |
| --- | --- | --- | --- | --- | --- | --- | --- | --- | --- |
| Outcome measures* | Estimate  (se) | Two-sided p-value | R^2^ | Estimate  (se) | Two-sided p-value | R^2^ | Estimate  (se) | Two-sided p-value | R^2^ |
| PedsQL physical functioning subscale | -1.39  (0.29) | <0.001 | 0.058 | -0.96  (0.26) | <0.001 | 0.050 | -1.39  (0.33) | <0.001 | 0.050 |

*Linear regression model was applied using PCSS-C/Y neurocognitive subscale as the predictor and PedsQL physical functioning subscale as criterion respectively, with age and gender inputted as covariates.

1. Olfactory subscale

|  | Self-report (n =33) | | | Parent-report (n = 27) | | |
| --- | --- | --- | --- | --- | --- | --- |
| Outcome measures* | Estimate  (se) | Two-sided p-value | R^2^ | Estimate  (se) | Two-sided p-value | R^2^ |
| SNOT | 0.31 (0.12) | 0.025 | 0.142 | 0.50 (0.14) | 0.021 | 0.336 |

*Linear regression model was applied using PCSS-C/Y olfactory subscale as the predictor and SNOT scale used as criterion, with age and gender inputted as covariates.

1. Non-specific somatic symptoms subscale

|  | Young adult self-report (n =278) | | | | Adolescent self-report (n =274) | | | | Parent-report (n = 245) | | | |
| --- | --- | --- | --- | --- | --- | --- | --- | --- | --- | --- | --- | --- |
| Outcome measures* | Estimate  (se) | Two-sided p-value | FDR- adjusted p-value | R^2^ | Estimate  (se) | Two-sided p-value | FDR- adjusted p-value | R^2^ | Estimate  (se) | Two-sided p-value | FDR- adjusted p-value | R^2^ |
| Self-perceived health status | -1.20  (0.14) | <0.001 | <0.001 | 0.201 | -1.26  (0.16) | <0.001 | <0.001 | 0.176 | -1.57  (0.21) | <0.001 | <0.001 | 0.217 |

*Linear regression model was applied using PCSS-C/Y cardiorespiratory subscale as the predictor and self-perceived health status as criterion, with age and gender inputted as covariates.

Supplementary Table S5. Correlations between PCSS-C/Y and PedsQL and SDQ

1. Young adult self-report PCSS-Y and PedsQL and SDQ

|  | PCSS-Y | Neurocognitive | Cardiorespiratory | Olfactory | Non-specific Somatic symptoms |
| --- | --- | --- | --- | --- | --- |
| PedsQL total | -0.63** | -0.63** | -0.30** | -0.23** | -0.61** |
| SDQ total | 0.46** | 0.40** | 0.21** | 0.16** | 0.43** |
| PedsQL physical | -0.54** | -0.46** | -0.28** | -0.21** | -0.55** |
| PedsQL emotional | -0.49** | -0.50** | -0.21** | -0.13* | -0.47** |
| PedsQL social | -0.44** | -0.43** | -0.21** | -0.25** | -0.42** |
| PedsQL school | -0.63** | -0.73** | -0.27** | -0.18** | -0.57** |
| SDQ Emotion | 0.40** | 0.37** | 0.16* | 0.09 | 0.37** |
| SDQ Conduct | 0.22** | 0.13* | 0.13* | 0.28** | 0.21** |
| SDQ Hyper | 0.33** | 0.40** | 0.12 | -0.02 | 0.30** |
| SDQ Peer | 0.23** | 0.1 | 0.16* | 0.14* | 0.22** |
| SDQ Prosocial | -0.01 | 0.07 | -0.02 | 0.00 | 0.00 |
| SDQ internal | 0.42** | 0.33** | 0.20** | 0.14* | 0.40** |
| SDQ external | 0.37** | 0.36** | 0.16* | 0.14* | 0.34** |

** Correlation is significant at the 0.01 level (two-tailed).

* Correlation is significant at the 0.05 level (two-tailed).

1. Adolescent self-report PCSS-Y and PedsQL and SDQ

|  | PCSS-Y | Neurocognitive | Cardiorespiratory | Olfactory | Non-specific Somatic symptoms |
| --- | --- | --- | --- | --- | --- |
| PedsQL total | -0.65** | -0.74** | -0.23** | -0.26** | -0.56** |
| SDQ total | 0.40** | 0.47** | 0.14* | 0.15* | 0.34** |
| PedsQL physical | -0.57** | -0.62** | -0.22** | -0.19** | -0.51** |
| PedsQL emotional | -0.58** | -0.62** | -0.21** | -0.27** | -0.53** |
| PedsQL social | -0.43** | -0.52** | -0.11 | -0.17** | -0.38** |
| PedsQL school | -0.63** | -0.81** | -0.25** | -0.26** | -0.52** |
| SDQ Emotion | 0.45** | 0.55** | 0.12 | 0.15* | 0.41** |
| SDQ Conduct | 0.14** | 0.17** | 0.11 | 0.08 | 0.1 |
| SDQ Hyper | 0.31** | 0.36** | 0.11 | 0.1 | 0.22** |
| SDQ Peer | 0.18** | 0.16* | 0.05 | 0.08 | 0.18** |
| SDQ Prosocial | 0.07 | 0.17** | -0.06 | 0.03 | 0.03 |
| SDQ internal | 0.42** | 0.48** | 0.11 | 0.15* | 0.39** |
| SDQ external | 0.28** | 0.33** | 0.14* | 0.11 | 0.20** |

** Correlation is significant at the 0.01 level (two-tailed).

* Correlation is significant at the 0.05 level (two-tailed).

1. Parent-report PCSS-C and PedsQL and SDQ

|  | PCSS-C | Neurocognitive | Cardiorespiratory | Olfactory | Non-specific Somatic symptoms |
| --- | --- | --- | --- | --- | --- |
| PedsQL total | -0.53** | -0.630** | -0.270** | -0.256** | -0.471** |
| SDQ total | 0.62** | 0.547** | 0.224** | 0.206** | 0.453** |
| PedsQL physical | -0.43** | -0.477** | -0.215** | -0.252** | -0.395** |
| PedsQL emotional | -0.53** | -0.512** | -0.276** | -0.210** | -0.491** |
| PedsQL social | -0.42** | -0.520** | -0.186** | -0.150** | -0.325** |
| PedsQL school | -0.55** | -0.746** | -0.284** | -0.272** | -0.458** |
| SDQ Emotion | 0.55** | 0.413** | 0.209** | 0.167** | 0.451** |
| SDQ Conduct | 0.40** | 0.344** | 0.116* | 0.147** | 0.304** |
| SDQ Hyper | 0.52** | 0.577** | 0.215** | 0.171** | 0.336** |
| SDQ Peer | 0.38** | 0.295** | 0.125* | 0.146** | 0.287** |
| SDQ Prosocial | -0.23** | -0.182** | -0.105* | -0.08 | -0.110* |
| SDQ internal | 0.56** | 0.423** | 0.201** | 0.185** | 0.444** |
| SDQ external | 0.54** | 0.544** | 0.197** | 0.182** | 0.364** |

** Correlation is significant at the 0.01 level (two-tailed).

* Correlation is significant at the 0.05 level (two-tailed).

Supplementary Table S6. Correlations between PCSS-C/Y and PedsQL and SDQ among infected cases confirmed by NP/ORF-8 antibody results.

|  | Self-report PCSS-Y (N= 84) | | | Parent-report PCSS-C (N= 69) | | | |
| --- | --- | --- | --- | --- | --- | --- | --- |
| Variables | Pearson R | Two-sided p-value | FDR-  adjusted p-value | | Pearson R | Two-sided p-value | FDR- adjusted p-value |
| PedsQL total score | -0.503 | <0.001 | <0.001 | | -0.675 | <0.001 | <0.001 |
| SDQ total score | 0.407 | <0.001 | <0.001 | | 0.563 | <0.001 | <0.001 |

PedsQL: Pediatric Quality of Life Inventory; SDQ: Strengths and Difficulties Questionnaire
* Linear regression model was applied using PCSS-C/Y total score as the predictor and PedsQL, SDQ and self-perceived health status as criterion, respectively, adjusted by age, gender and SES. Partial R-square (R^2^) was reported.

Supplementary Table S7. Cut-off scores for PCSS-C/Y with reference to mean PedsQL score

| Percentile rank | Young adult self-report PCSS-Y | Mean PedsQL score (SD) | Adolescent self-report PCSS-Y | Mean PedsQL score (SD) | Parent-report PCSS-C | Mean PedsQL score (SD) |
| --- | --- | --- | --- | --- | --- | --- |
| > 50^th^ percentile | > 22 | 66.58 (14.84) | > 18 | 65.71 (18.86) | > 15 | 72.37 (13.78) |
| > 90^th^ percentile (moderate risk) | > 37 | 58.92 (17.90) | > 37 | 57.67 (17.76) | > 30 | 56.79 (13.00) |
| > 95^th^ percentile (high risk) | > 40 | 55.30 (18.02) | > 41 | 55.87 (19.05) | > 36 | 50.50 (13.70) |

PedsQL: Pediatric Quality of Life Inventory


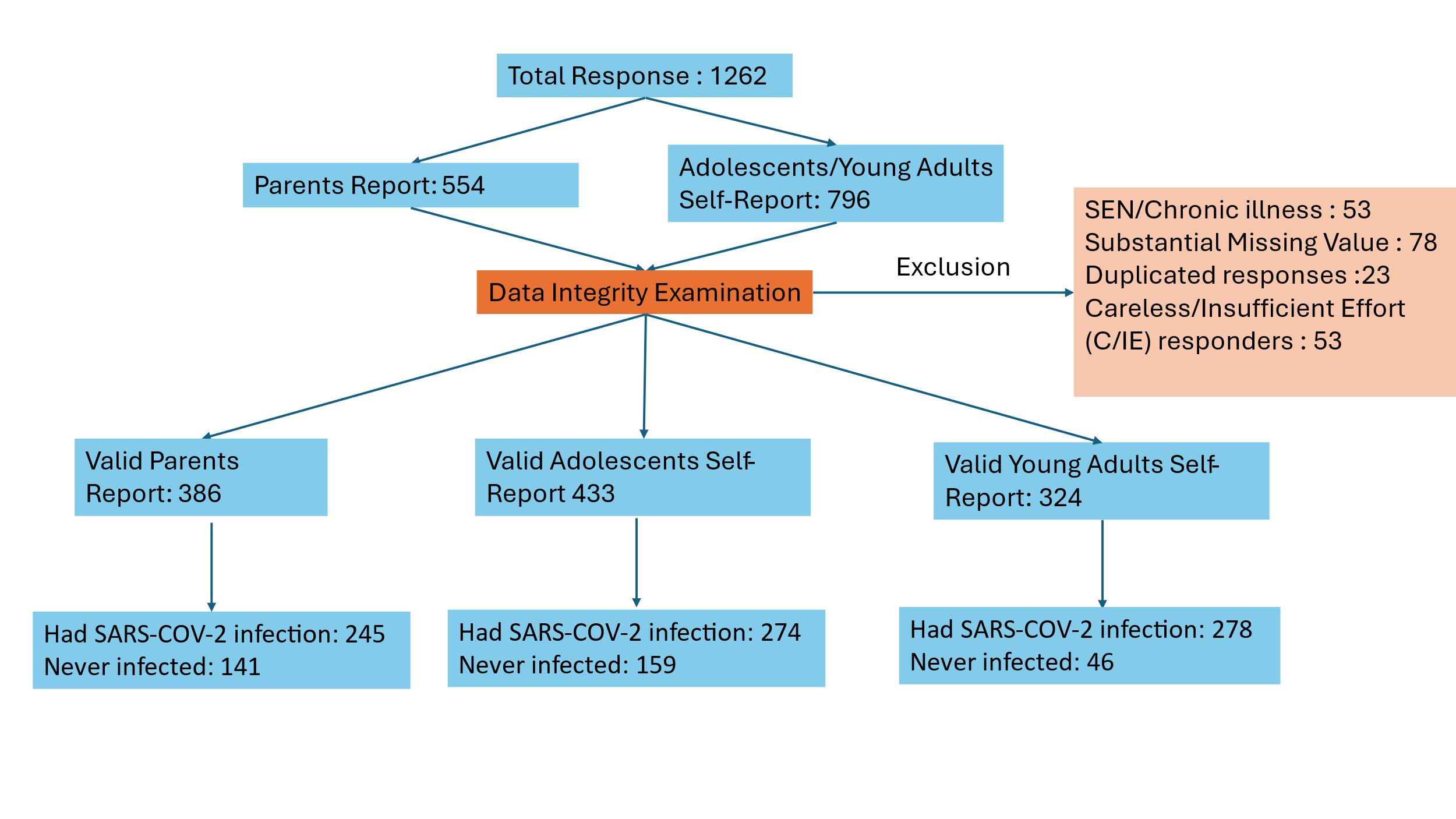


Figure S1a. Data inclusion flow chart.


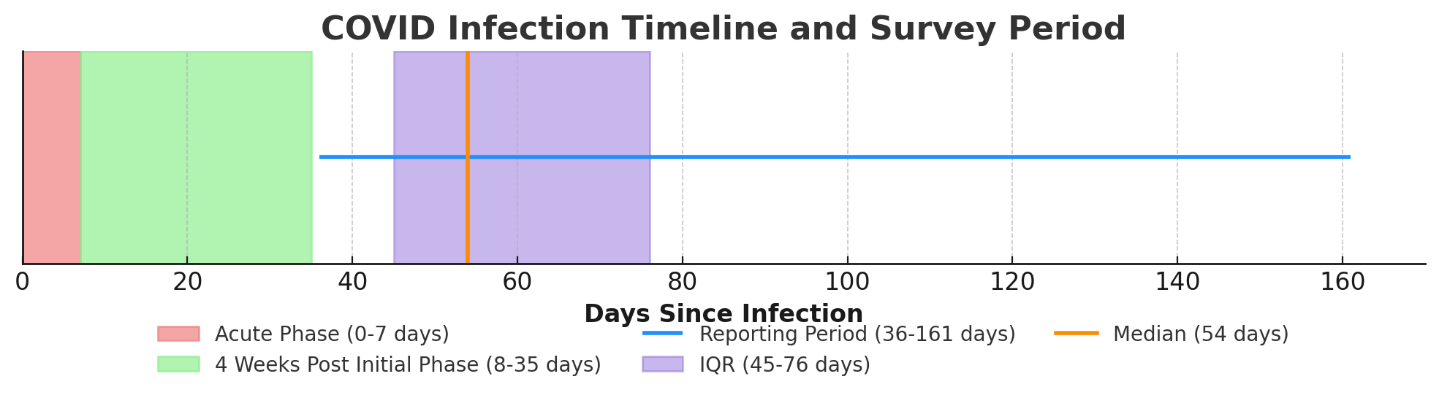


Figure S1b. The timeline of infection and survey participation for individuals infected with COVID-19 in this study


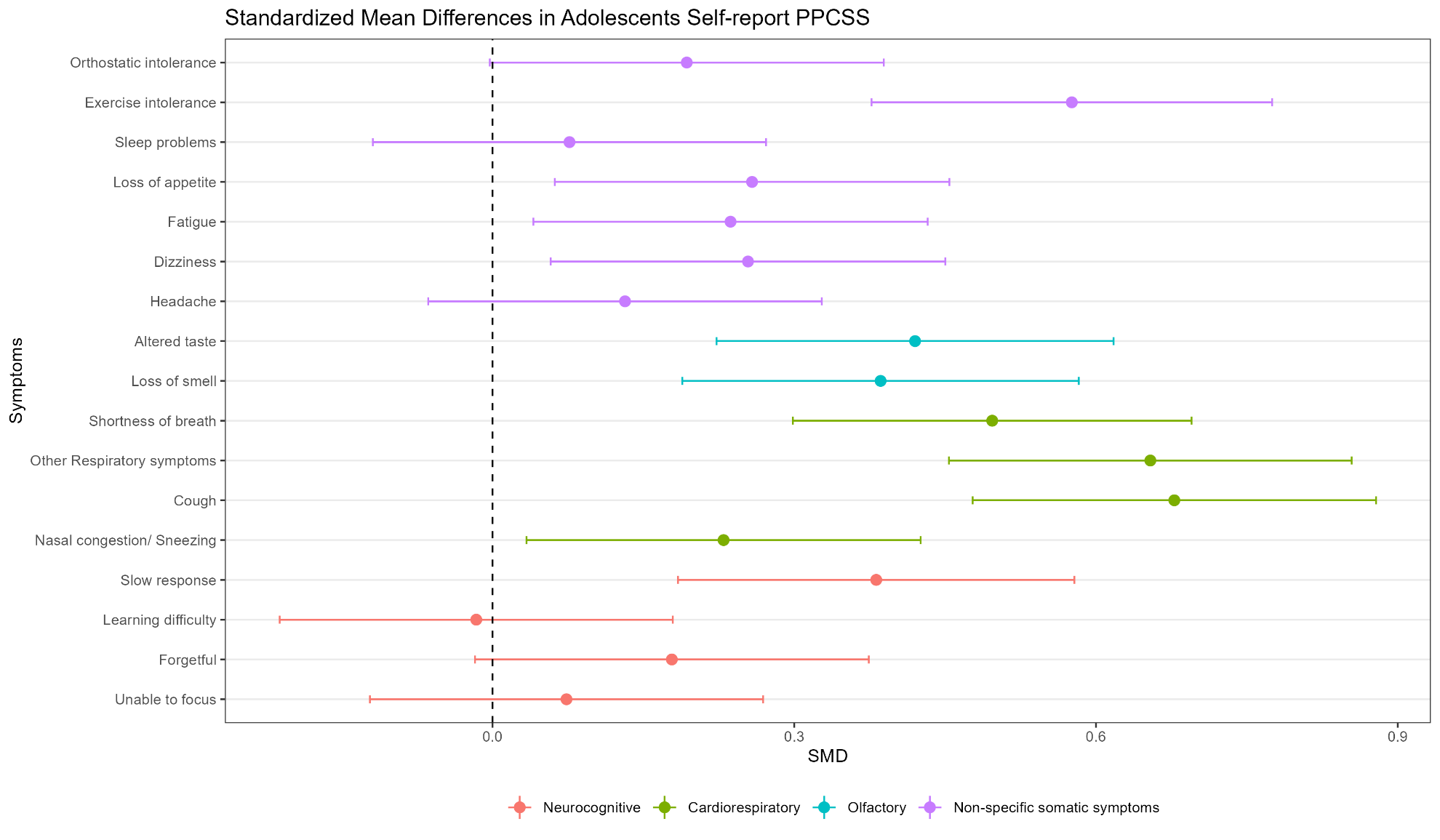


Figure S2a. Standardized Mean Differences in Adolescent Self-report PPCS. Error bars represent 95% CI.


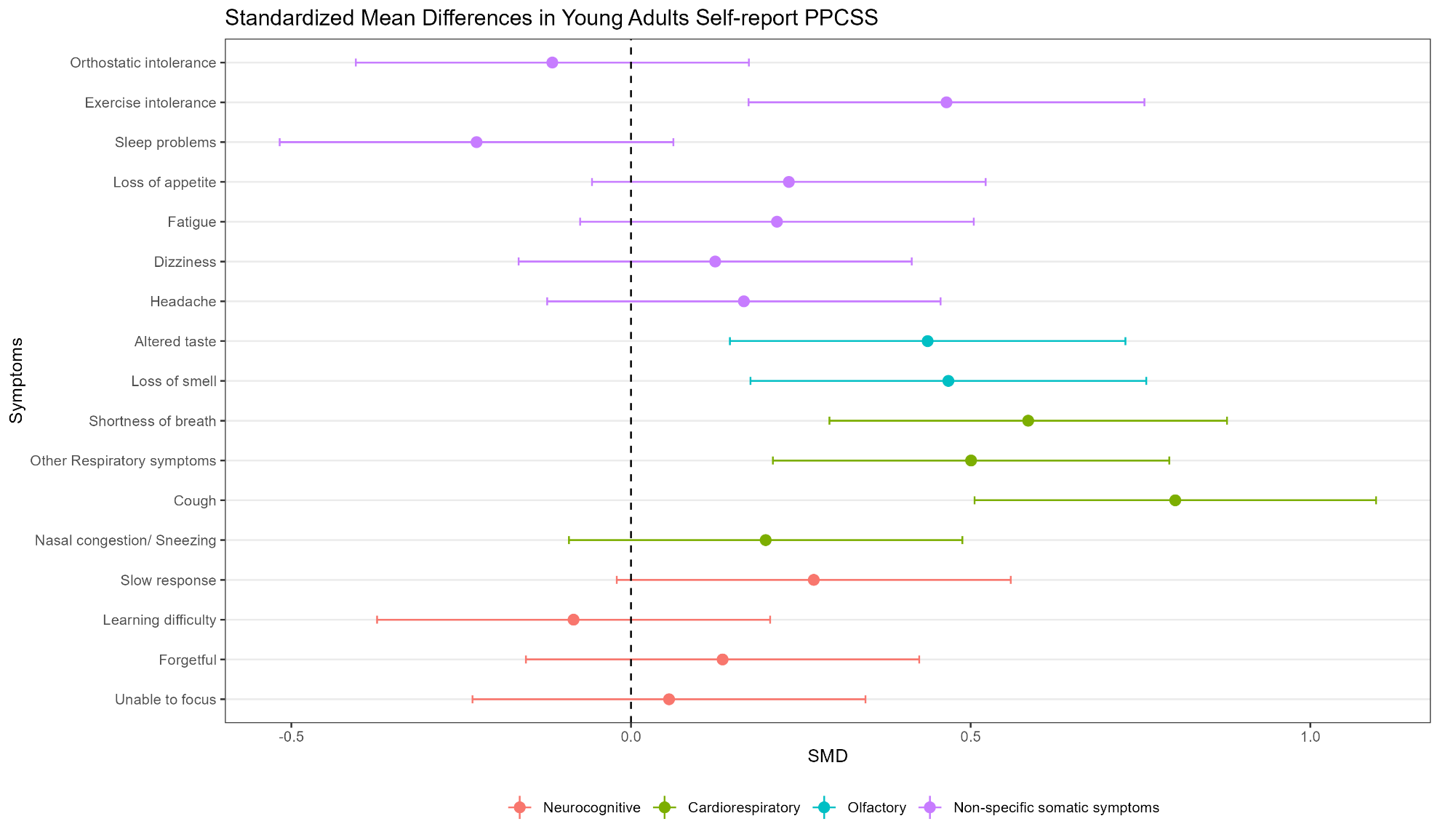


Figure S2b. Standardized Mean Differences in Young Adult Self-report PCSS-Y. Error bars represent 95% CI.


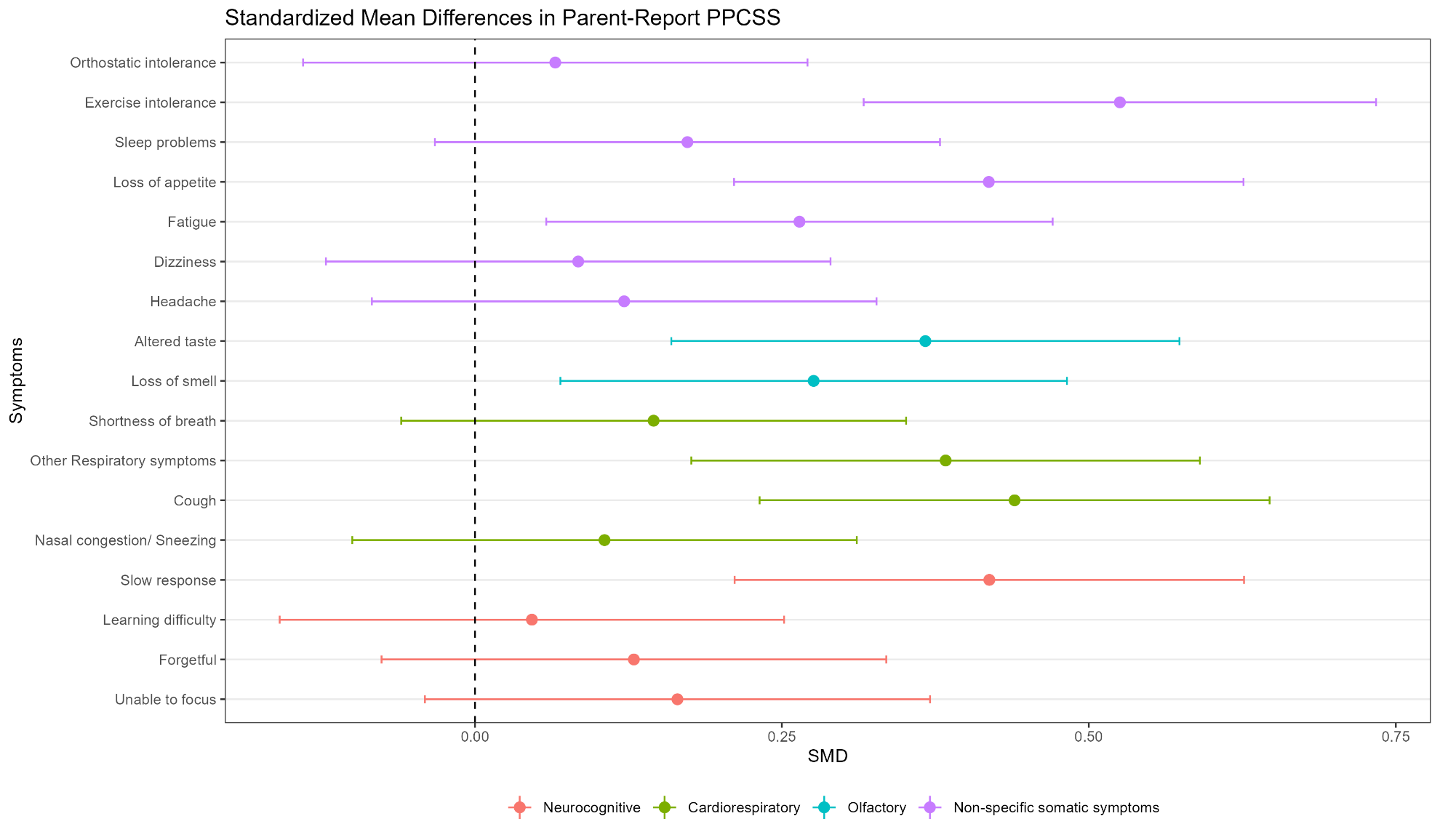


Figure S2c. Comparison of PCSS-C symptoms in parent-reported children and adolescents infected with SARS-CoV-2 and control subjects. Error bars represent 95% CI.


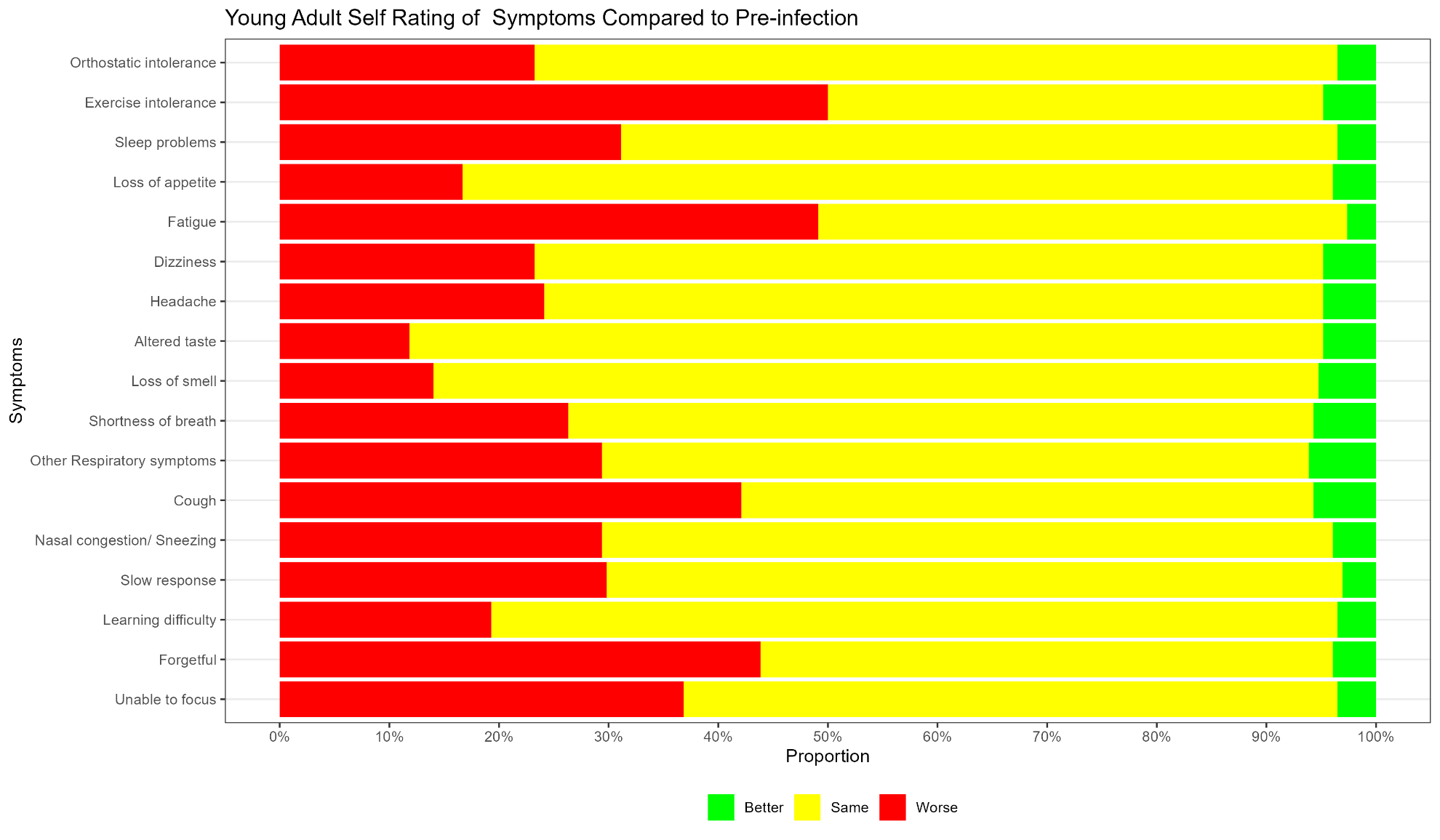


Figure S3a. Young adult self-Rating post-COVID symptoms compared to pre-infection.


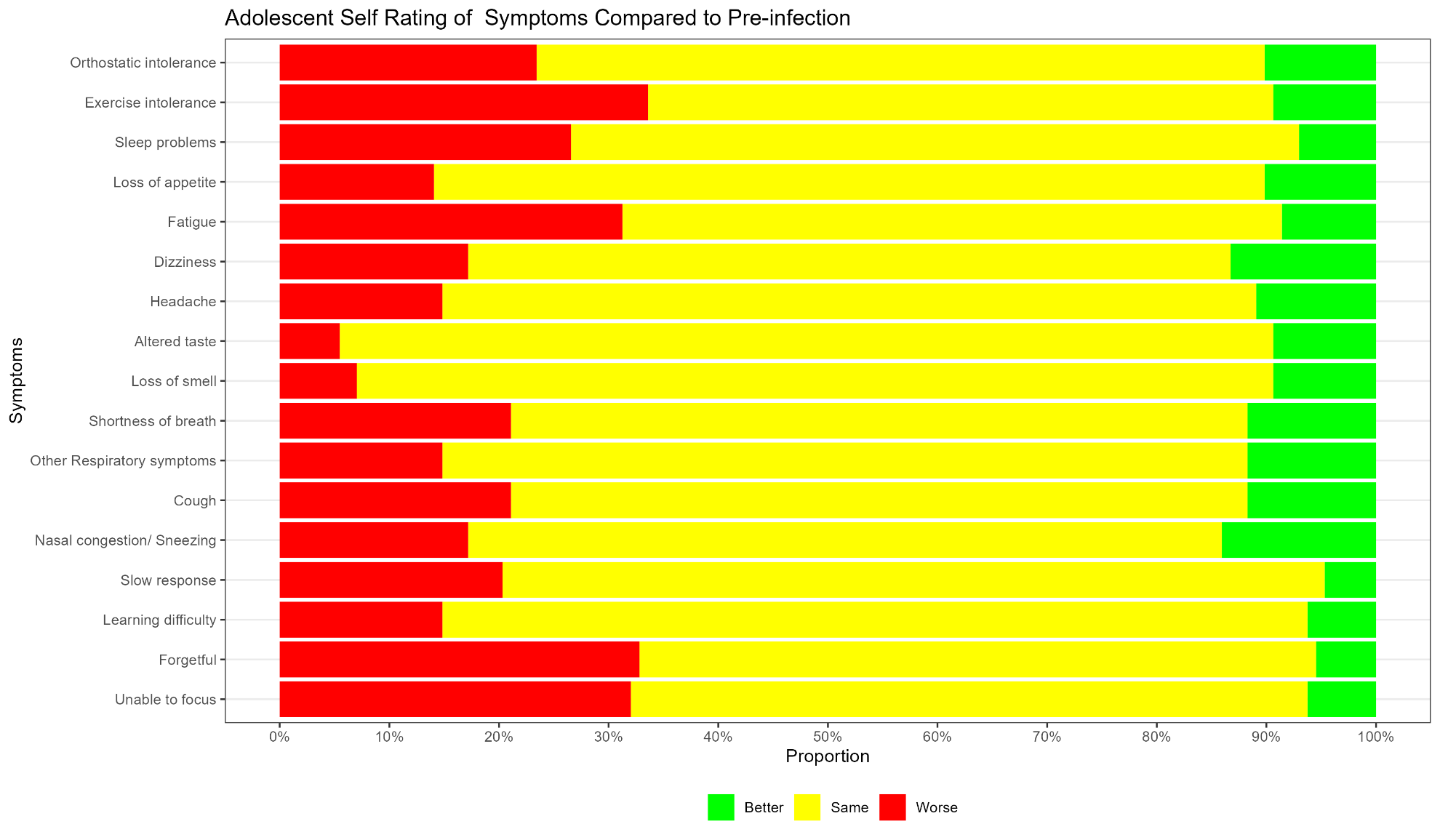


Figure S3b. Adolescent self-Rating post-COVID symptoms compared to pre-infection.


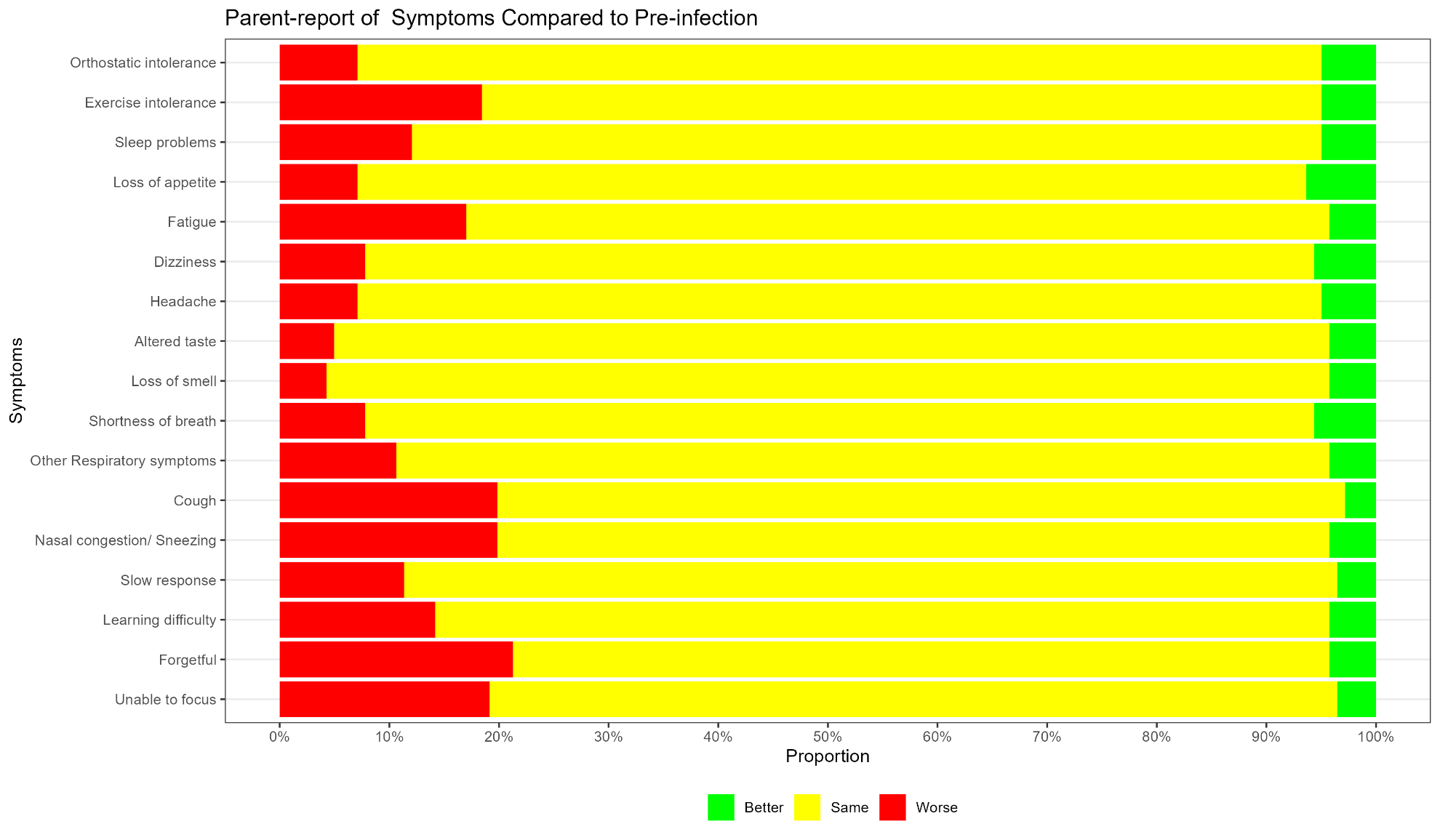


Figure S3c. Parent-reported post-COVID symptoms compared to pre-infection.


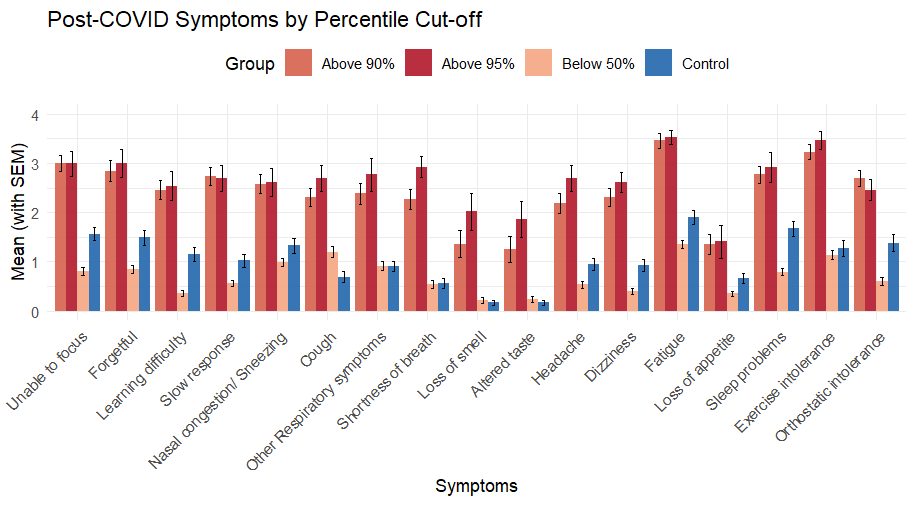


Figure S4a. Young adult self-Rating post-COVID symptoms by percentile cut-offs.


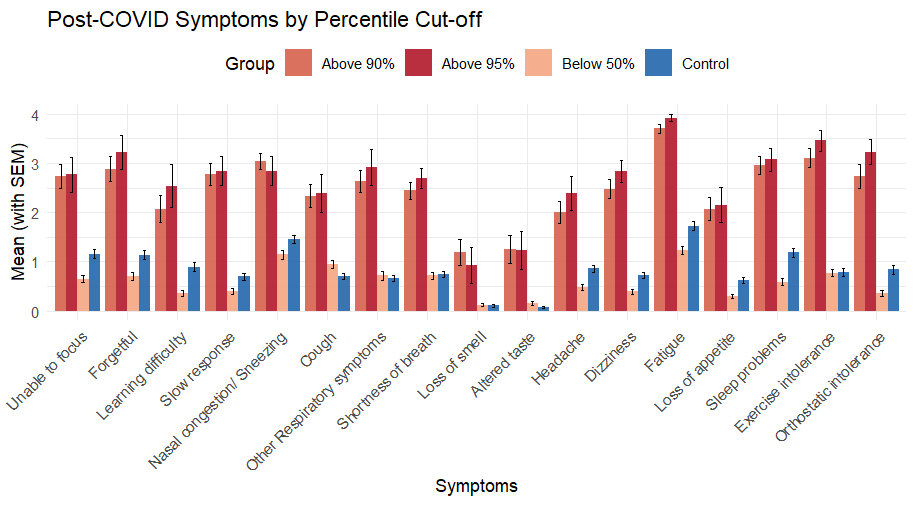


Figure S4b. Adolescent self-Rating post-COVID symptoms by percentile cut-offs.


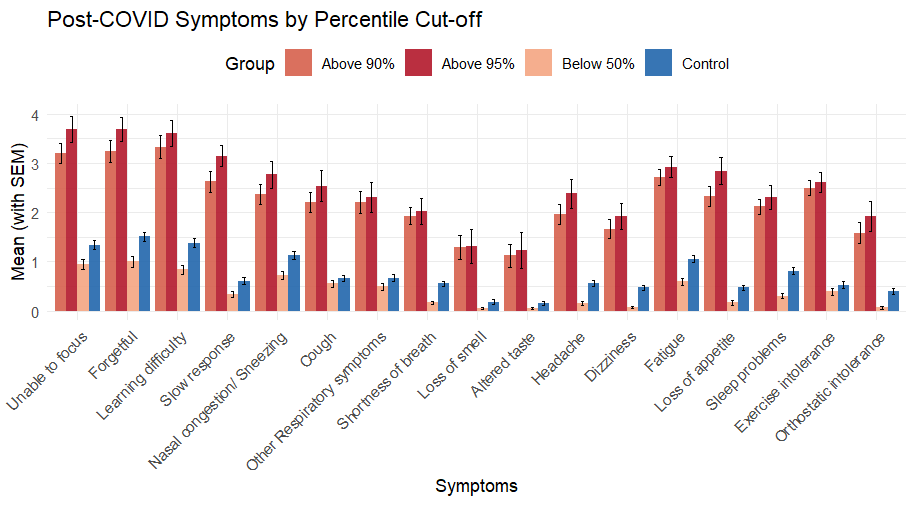


Figure S4C. Parent-report post-COVID symptoms by percentile cut-offs.
