# Supplementary material for: Development and validation of the Post-COVID Symptom Scale for Children/Youth (PCSS-C/Y)
Source: Eur J Pediatr. 2024 Dec 13;184(1):81. doi: 10.1007/s00431-024-05913-9 (PMC11645425; doi:10.1007/s00431-024-05913-9)

The development of the Post-COVID Symptom Scale for Children/Youth (PCSS-C/Y) began with an extensive literature review and international consensus, identifying a comprehensive pool of 62 symptoms potentially associated with post-COVID conditions in children. The expert panel then reviewed this initial pool to determine the most prevalent and relevant symptoms for the paediatric population.

The initial symptoms pool included:

- **Mood and Psychological**: Sad, tense, angry, depression, anxiety.
- **Fatigue**: Fatigue.
- **Sleep Disorders**: Insomnia, hypersomnia, poor sleep quality.
- **Headache**: Headache.
- **Respiratory Symptoms**: Shortness of breath, chest tightness, chest pain, wheezing, cough, sputum production, nasal congestion/sneezing.
- **Cognitive Issues**: Less concentration (unable to focus), learning difficulties, confusion, memory loss (forgetful), slow response.
- **Loss of Appetite**: Loss of appetite.
- **Body Weight Changes**: Weight loss.
- **Exercise Intolerance**: Exercise intolerance.
- **Altered Smell and Taste**: Altered (loss) smell, hyposmia, anosmia, parosmia, phantom smell, altered taste
- **Sweating**: Hyperhidrosis.
- **Dizziness**: Dizziness, vertigo.
- **Rhinorrhea**: Rhinorrhea.
- **Myalgia/Arthralgia**: Myalgia, arthralgia.
- **Ophthalmologic Issues**: Conjunctivitis, dry eye, blurred vision, photophobia, eye pain.
- **Gastrointestinal Symptoms**: Abdominal pain, nausea, vomiting, diarrhea, constipation.
- **Dermatologic Symptoms**: Dry skin, itchy skin, rashes, hives, hair loss.
- **Sore Throat**: Sore throat.
- **Palpitations**: Palpitations.
- **Speech Disturbances**: Dysphonia.
- **Neurological Abnormalities**: Pins and needles, tremor, numbness.
- **Urinary Symptoms**: Urinary problems.
- **Dysphagia**: Dysphagia.
- **Miscellaneous**: Chills, sweats, hot flushes, sicca syndrome, red eye, eye irritation.

This review process resulted in the selection of 20 items for the initial PCSS-C/Y, further grouping them into *Brain fog/ neurocognitive symptoms, Neuropsychiatric symptoms, Olfactory symptoms and Non-specific* somatic symptoms. These items were then subjected to validity testing within the current sample. Following the testing, it was determined that there were no significant differences in the neuropsychiatric measures across the sub-cohorts. Consequently, three items related to neuropsychiatric symptoms were excluded from the PCSS-C/Y, resulting in the current 17-item scale.


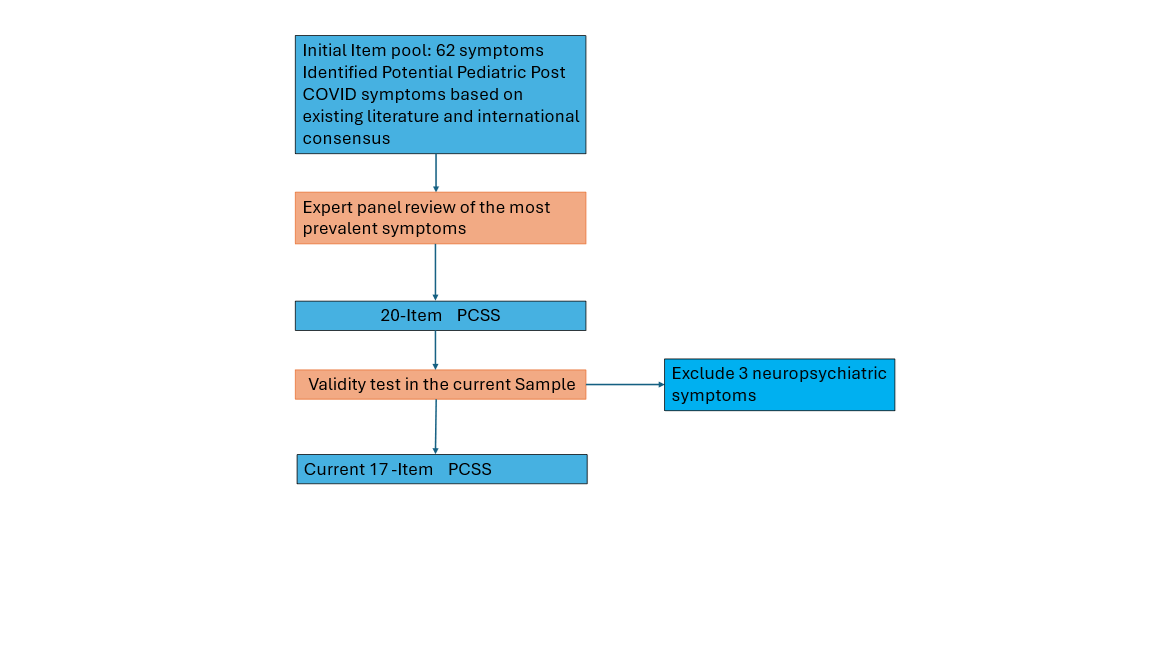

Supplement: Supplementary file 2 — Supplementary file2 (DOCX 51 KB) [file 431_2024_5913_MOESM2_ESM.docx]
