# Supplementary material for: Development and validation of the Post-COVID Symptom Scale for Children/Youth (PCSS-C/Y)
Source: Eur J Pediatr. 2024 Dec 13;184(1):81. doi: 10.1007/s00431-024-05913-9 (PMC11645425; doi:10.1007/s00431-024-05913-9)
Supplement: Supplementary file 3 — Supplementary file3 (DOCX 28 KB) [file 431_2024_5913_MOESM3_ESM.docx]

Supplementary file 3: the 20 item PCSS-C/Y-20

Supplementary Table S3.1. PCSS-C/Y-20 items

| PCSS-C/Y-20 subscales | Items in English | Items in Chinese |
| --- | --- | --- |
| Brain fog/ neurocognitive symptoms | Unable to focus, being easily distracted | 專注力弱,容易分心,不能長 |
|  | Memory issue, forgetful | 記憶力問題、善忘 |
|  | Learning difficulty, cannot keep up with homework | 學習問題,功課跟不上 |
|  | Slow response | 反應較慢 |
|  |  |  |
| Cardiorespiratory symptoms | Nasal congestion/ Sneezing | 鼻塞/ 流鼻水 |
|  | Other Respiratory symptoms (e.g., sore throat, sputum) | 其他上呼吸道症狀(例如:喉嚨痛、有痰) |
|  | Shortness of breath | 呼吸困難/氣促 |
|  | Cough | 咳嗽 |
| Neuropsychiatric symptoms | Feeling sad | 感到傷心 |
|  | Feeling anxious | 感到焦慮 |
|  | Feeling nervous/stressed | 感到緊張/壓力大 |
| Olfactory symptoms | Loss of smell | 嗅覺失靈 |
|  | Altered taste | 味覺失靈 |
| Non-specific somatic symptoms | Loss of appetite | 食慾不振 |
|  | Headache | 頭痛 |
|  | Dizziness | 頭暈 |
|  | Sleep problems (e.g., Insomnia, hypersomnia) | 睡眠問題(例如失眠或嗜睡) |
|  | Exercise intolerance | 體能變差/易攰 |
|  | Orthostatic intolerance (dizziness, weakness or nausea when standing up) | 自律神經失調(蹲下久坐起身時頭暈) |
|  | Fatigue | 疲倦 |

Table S3.2. Internal Consistency of The PCSS-C/Y-20.

|  | Self-report PCSS-C/Y-20 | | Parent-report PCSS-C/Y-20 |
| --- | --- | --- | --- |
|  | Young adults (N=278) | Adolescents  (N =274) | Children and adolescents  (N= 245) |
| Neurocognitive | 0.89 | 0.85 | 0.89 |
| Cardiorespiratory | 0.76 | 0.80 | 0.82 |
| Neuropsychiatric | 0.85 | 0.83 | 0.79 |
| Olfactory | 0.88 | 0.81 | 0.93 |
| Non-specific Somatic symptoms | 0.86 | 0.86 | 0.89 |
| Whole scale | 0.91 | 0.91 | 0.92 |

| Table S3.3. Comparison of PCSS-C/Y-20 scores of infected cases and control subjects | | | | | | | | | | | | |
| --- | --- | --- | --- | --- | --- | --- | --- | --- | --- | --- | --- | --- |
|  | Young adult self-report PCSS-C/Y-20 (278 vs. 46) | | | | Adolescent self-report PCSS-C/Y-20  (274 vs. 159) | | | | Parent-report PCSS-C/Y-20  (245 vs. 141) | | | |
|  | t | Two-sided p-value | FDR-adjusted p-value | Cohen's d | t | Two-sided p-value | FDR-adjusted  p-value | Cohen's d | t | Two-sided p-value | FDR-adjusted p-value | Cohen's d |
| Neurocognitive | 2.067 | 0.042 | 0.050 | 0.27 | 2.009 | 0.045 | 0.054 | 0.19 | 1.753 | 0.081 | 0.097 | 0.20 |
| Cardiorespiratory | 6.278 | <0.001 | <0.001 | 0.80 | 7.219 | <0.001 | <0.001 | 0.65 | 4.063 | <0.001 | <0.001 | 0.37 |
| Neuropsychiatric | -0.290 | 0.773 | 0.773 | -0.04 | 0.262 | 0.793 | 0.793 | 0.03 | -0.761 | 0.447 | 0.447 | -0.10 |
| Olfactory | 5.820 | <0.001 | <0.001 | 0.50 | 5.326 | <0.001 | <0.001 | 0.44 | 3.158 | 0.002 | 0.003 | 0.31 |
| Non-specific somatic symptoms | 2.472 | 0.01 | 0.002 | 0.39 | 3.631 | <0.001 | <0.001 | 0.35 | 3.735 | <0.001 | 0.001 | 0.39 |
| Whole PCSS-C/Y-20 | 3.163 | 0.02 | 0.004 | 0.50 | 4.681 | <0.001 | <0.001 | 0.44 | 3.232 | 0.001 | 0.003 | 0.34 |

| Table S3.4. Construct validity of PCSS-C/Y-20 (whole scale) | | | | | | | | | | | | |
| --- | --- | --- | --- | --- | --- | --- | --- | --- | --- | --- | --- | --- |
| Young adult self-report PCSS-C/Y-20 (n =278) | | | | | Adolescent self-report PCSS-C/Y-20  (n =274) | | | | Parent-report PCSS-C/Y-20 (n = 245) | | | |
|  | Estimate  (se) | Two-sided p-value | FDR-adjusted q-value | R^2^ | Estimate  (se) | Two-sided p-value | FDR-adjusted q-value | R^2^ | Estimate  (se) | Two-sided p-value | FDR-adjusted q-value | R^2^ |
| PedsQL total score | -0.75  (0.06) | <0.001 | <0.001 | 0.394 | -0.76  (0.07) | <0.001 | <0.001 | 0.303 | -0.83  (0.05) | <0.001 | <0.001 | 0.547 |
| SDQ total score | 0.18  (0.02) | <0.001 | <0.001 | 0.239 | 0.22  (0.02) | <0.001 | <0.001 | 0.246 | 0.29  (0.02) | <0.001 | <0.001 | 0.324 |
| Self-perceived health status | -0.48  (0.05) | <0.001 | <0.001 | 0.204 | -0.62  (0.07) | <0.001 | <0.001 | 0.223 | -0.70  (0.07) | <0.001 | <0.001 | 0.286 |
| *Linear regression model was applied using PCSS-C/Y-20 total score as the predictor and PedsQL SDQ and self-perceived health status as criterion respectively, with age gender and SES inputted as covariates. | | | | | | | | | | | | |

Table S3.5. Cut-off scores for PCSS-C/Y-20.

| Percentile rank | Young adult self-report PCSS-C/Y-20 | Mean PedsQL score (SD) | Adolescent self-report PCSS-C/Y-20 | Mean PedsQL score (SD) | Parent-report PCSS-C/Y-20 | Mean PedsQL score (SD) |
| --- | --- | --- | --- | --- | --- | --- |
| > 50% | >25 | 65.67(15.04) | >25 | 63.42(17.98) | >17 | 72.04 (13.44) |
| > 90%  =moderate risk | >43 | 60.08(17.68) | >43 | 50.74(14.99) | >33 | 57.21 (13.45) |
| > 95%  =high risk | >47 | 52.27(15.92) | >47 | 45.74(12.59) | >39 | 49.32 (9.78) |
|  |  |  |  |  |  |  |

| Table S3.6. Construct validity of Neuropsychiatric subscale of PCSS-C/Y-20 | | | | | | | | | | | | |
| --- | --- | --- | --- | --- | --- | --- | --- | --- | --- | --- | --- | --- |
|  | Young adult self-report PCSS-C/Y-20 Neuropsychiatric subscale (n =278) | | | | Adolescent self-report PCSS-C/Y-20 Neuropsychiatric subscale  (n =274) | | | | Parent-report PCSS-C/Y-20 Neuropsychiatric subscale (n = 245) | | | |
| Outcome measures* | Estimate  (se) | Two-sided p-value | FDR- adjusted p-value | R^2^ | Estimate  (se) | Two-sided p-value | FDR- adjusted p-value | R^2^ | Estimate  (se) | Two-sided p-value | FDR- adjusted p-value | R^2^ |
| PedsQL emotional subscale | -5.07  (0.37) | <0.001 | <0.001 | 0.418 | -4.61  (0.0.35) | <0.001 | <0.001 | 0.452 | -3.54  (0.37) | <0.001 | <0.001 | 0.322 |
| DASS | 0.12 (0.01) | <0.001 | <0.001 | 0.340 | 0.11  (0.02) | <0.001 | <0.001 | 0.392 | NA | | | |
| SDQ emotional subscale | 0.46  (0.04) | <0.001 | <0.001 | 0.312 | 0.57 (0.35) | <0.001 | <0.001 | 0.211 | 0.58  (0.04) | <0.001 | <0.001 | 0.513 |
| *Linear regression model was applied using PCSS-C/Y-20 neuropsychiatric subscale as the predictor, PedsQL emotional subscale,  DASS total score, and SDQ emotional subscale as criterion, respectively, with age and gender inputted as covariates. | | | | | | | | | | | | |
